# Multi-population GWAS detects robust marker associations in a newly established six-rowed winter barley breeding program

**DOI:** 10.1038/s41437-024-00733-x

**Published:** 2024-11-28

**Authors:** Cathrine Kiel Skovbjerg, Pernille Sarup, Ellen Wahlström, Jens Due Jensen, Jihad Orabi, Lotte Olesen, Just Jensen, Ahmed Jahoor, Guillaume Ramstein

**Affiliations:** 1 Nordic Seed A/S, Odder, Denmark; 2 Center for Quantitative Genetics and Genomics, Aarhus University, Aarhus C, Denmark; 3 Department of Plant Breeding, The Swedish University of Agricultural Sciences, Alnarp, Sweden

**Keywords:** Agricultural genetics, Population genetics, Genome-wide association studies, Rare variants, Plant breeding

## Abstract

Genome-wide association study (GWAS) is a powerful tool for identifying marker-trait associations that can accelerate breeding progress. Yet, its power is typically constrained in newly established breeding programs where large phenotypic and genotypic datasets have not yet accumulated. Expanding the dataset by inclusion of data from well-established breeding programs with many years of phenotyping and genotyping can potentially address this problem. In this study we performed single- and multi-population GWAS on heading date and lodging in four barley breeding populations with varying combinations of row-type and growth habit. Focusing on a recently established 6-rowed winter (6RW) barley population, single-population GWAS hardly resulted in any significant associations. Nevertheless, the combination of the 6RW target population with other populations in multi-population GWAS detected four and five robust candidate quantitative trait loci for heading date and lodging, respectively. Of these, three remained undetected when analysing the combined populations individually. Further, multi-population GWAS detected markers capturing a larger proportion of genetic variance in 6RW. For multi-population GWAS, we compared the findings of a univariate model (MP1) with a multivariate model (MP2). While both models surpassed single-population GWAS in power, MP2 offered a significant advantage by having more realistic assumptions while pointing towards robust marker-trait associations across populations. Additionally, comparisons of GWAS findings for MP2 and single-population GWAS allowed identification of population-specific loci. In conclusion, our study presents a promising approach to kick-start genomics-based breeding in newly established breeding populations.

## Introduction

Barley (*Hordeum Vulgare L.)* is the fourth most produced cereal crop in the world. In 2021 its global production was 156 million tonnes with the majority (>60%) originating from European countries (FAOSTAT 2023). As barley is used for both animal feed and malt production and cultivated both as a winter and a spring crop, different market and consumer preferences exist leading to different breeding programs maintained at breeding companies (Charmet et al. 2023). An example of different breeding populations is six-rowed and two-rowed barley that morphologically differ in the number of grain-producing spikelets and grain size (Koppolu et al. 2013). Another example is the spring versus winter cultivars which differ in their optimal growing season due to different vernalization requirements and flowering times (Distelfeld et al. 2009; Fernández-Calleja et al. 2021; Sasani et al. 2009). Historically, these breeding populations are rarely mixed, are generated from different founders, and have experienced different selection and drift. Consequently, they are genetically differentiated in more genes than those associated with vernalization, flowering time and row-type (Bengtsson et al. 2017; Bustos‐Korts et al. 2019; Hamblin et al. 2010; Pauli et al. 2014).

Determining the genetic architecture of key agronomic traits is important for crop improvement. Genome-wide association studies (GWAS) are common approaches to study the additive genetics underlying traits and have successfully pointed to genomic regions associated with agronomic traits e.g. plant height and heading date in cultivated barley (Alqudah et al. 2016; Pasam et al. 2012; Pauli et al. 2014; Wang et al. 2012). A successful GWAS requires sufficient genetic variation for the trait of interest. This has traditionally been obtained by using diversity panels of largely unrelated individuals. However, there are several important benefits of applying GWAS to populations from applied breeding programs instead. First, findings are directly applicable to the studied breeding program, as no identified quantitative trait loci (QTLs) are already fixed by breeding (Würschum 2012; Quero et al. 2018). Another benefit is that breeding populations are typically highly adapted to the environments where they are cultivated and phenotyped. In barley, breeding populations have already proven applicable in detection of QTLs for many traits including heading date and lodging (Pauli et al. 2014; Tsai et al. 2020).

Comparing GWAS findings from multiple populations may be useful for reliable inference of additive genetic effects. Previous studies have used QTL colocalization analysis to identify conserved as well as population-specific QTLs in several species (Tao et al. 2020; Thareja et al. 2023). Another main reason for studying multiple breeding populations is that older breeding populations with many years of routine phenotyping contain genetic information that potentially can be transferred to the smaller and more recently established populations. This is important, as adaptation to new markets and changing climates can call for new breeding programs. To ensure genetic progress in these programs, application of genetic tools, including GWAS, is important. However, sample size is a major limiting factor of the statistical power to detect signals in GWAS (Sham and Purcell 2014). Combining populations for GWAS has earlier proved a good strategy for detecting signals that remain undiscovered when studying the individual populations (Gebreyesus et al. 2019; Zuffo et al. 2022). Further, taking advantage of different linkage disequilibrium (LD) patterns in different populations can be helpful in fine-mapping QTLs (Rosenberg et al. 2010).

Yet, combining different breeding populations in a joint GWAS analysis comes with challenges, as these populations are typically not designed for joint analysis (Wallace et al. 2016; Quero et al. 2018). Heterogeneity between populations can result from the populations being measured in different environments and/or growing seasons, the populations exhibiting different LD patterns, the phase of SNP and QTL alleles being reversed among the populations due to recombination events, differences in interactions between QTL and environments, and different interactions between QTL and the genetic background i.e. epistasis (Begum et al. 2012; Guillenea et al. 2022; Karaman et al. 2021). Thus, it cannot be assumed that SNP effects are the same across populations, but rather partly correlated (Karaman et al. 2021; Legarra et al. 2021). To date, most multi-population association studies in plants have been performed by adjusting for differences in environmental and population means. Although loosening the extreme assumption of a trait being genetically independent between populations, as assumed when analysing populations separately, the assumption of a trait being genetically identical between populations still remains (Alqudah et al. 2016; Müller et al. 2019; Zuffo et al. 2022). Therefore, GWAS models that allow for partial genetic correlations between the same trait measured in different populations i.e. multivariate models, make more realistic assumptions. In fact, studies on genomic prediction across populations have demonstrated that using a multivariate model to address trait genetic heterogeneity across populations yields more accurate results than a univariate model (De Haas et al. 2012; Lehermeier et al. 2015; Olson et al. 2012).

Here, we focus on a recently established Nordic Seed A/S breeding population of 6-rowed winter (6RW) barley. It was created to meet the market interest in hybrid barley populations and consequently consists of parental hybrid components. Among important breeding goal traits are synchronized flowering of male and female parental components, making heading date a key trait. Like most other barley breeding populations, another important trait is stem lodging resistance, as lodging largely reduces grain yield, quality, and complicates harvesting (Rajkumara 2008). Because of its recent establishment in 2018, little phenotype and genotype data has been accumulated in the 6RW population so far. Nevertheless, additional datasets from three other barley breeding programs that differ in their combinations of row (two-rowed versus six-rowed) and growth type (winter versus spring), are available for analysis. This study aims to (1) investigate the genetic diversity between the 6RW and the remaining barley breeding populations, (2) develop suitable GWAS models that successfully combine 6RW with other populations to increase detection power and precision, and (3) identify conserved and population-specific QTLs associated with the studied traits.

## Materials and methods

### Populations and plant material

Four breeding populations, collectively comprising 5805 inbred barley lines, were included in this study. The populations originate from Nordic Seed A/S and differ in their combination of growth habit (winter versus spring) and row-type (2-rowed versus 6-rowed). Further information on the populations can be found in Table 1. In summary, the 2-rowed spring population (2RS) comprised 3346 lines, the 2-rowed winter (2RW) population comprised 1501 lines, the 6RW population comprised 511 lines, and the 6-rowed spring (6RS) population comprised 447 lines. All stated population sizes are reported after outlier removal as described in the section on principal component analysis (PCA).

**Table 1:** Characteristics of the four breeding populations.

	Population			
	6RW	2RW	6RS	2RS
Row type	Six	Two	Six	Two
Growth habit	Winter	Winter	Spring	Spring
Number of genotypes	511	1501	447	3346
Number of full-sib families	57	282	85	1255
Method of inbreeding	DH	DH	Selfing with SSD in F4^a^	Selfing with SSD in F4^a^
First genotyped crossings^b^	2018	2011	2015	2005
Number of polymorphic markers	11811	11711	10862	11748

*DH* double haploid technique, *SSD* single seed descent.

^a^Spring lines were genotyped at F5 and phenotyped at F6, with some selected lines phenotyped at F7.

^b^Year of crossing for earliest genotyped lines. For all populations, except 2RS, this is also the year breeding programs were established i.e. breeding activities were initiated. The 2RS breeding program has been running for more than 40 years.

### Field experiments and phenotypic data

All populations were phenotyped for heading date and lodging. The number of phenotyped plots varied by population and trait. In total, the phenotypic data included growing seasons from 2013 to 2023, and a set of 16 geographical locations representing fields in Denmark, Germany, Finland, and France (Table S1). Each year, new crosses were introduced and tested, while some older lines were discontinued. Consequently, the set of lines differed between years with a degree of overlap between adjacent years. Testing was done annually in multiple locations. Within each combination of year-location, genotypes were organized in trials in an alpha-lattice design with 2–3 replicates. Plants were grown in plots with sizes of 10–15 m^2^. *X* and *Y* field coordinates for each plot were noted within each trial and used to allow for modelling spatial effects by moving average over adjacent plots within trials.

The heading date of a plot was measured as the date where 50% of the main spikes (the first spike of the plant) had 1–2 cm of visible awns protruding the flag leaf (BBCH scale 49–51). Heading dates were recorded as days starting from May 1st. Stem lodging was visually scored on a scale from 1–9, where 9 is most severe. Further details can be found in Table S2.

### Genotyping, imputation and SNP filtration

DNA was extracted from 2-week-old seedlings using a Cetyl Trimethyl Ammonium Bromide method as described by Orabi et al. (2014). Individuals genotyped after 2013 were genotyped with the iSelect Illumina Infinium 15 K SNP chip, remaining individuals were genotyped with the iSelect Illumina Infinium 9 K SNP array. Genotyping by both arrays was outsourced to TraitGenetics GmbH (Gatersleben, Germany). Genotypes with the 15 K SNP were used as a reference for within-population imputation of the 9 K genotypes. Imputation was performed using Beagle v.5.4 with default settings and a sliding window of 150 cM. The imputation accuracy was tested within populations using a cross-validation (CV) scheme where the genotypes of *q* random individuals were set to missing at the 15 K chip-specific SNPs. The parameter *q* was set so that it fitted the number of genotypes to predict in the 9 K array. The procedure was repeated 100 times. An average imputation concordance of 0.97–0.98 was achieved. Prior to imputation, a SNP call rate filter of >0.8 was applied to a larger set of both unphenotyped and phenotyped individuals (6RW = 1668; 2RW = 5431; 6RS = 945; 2RS = 17,732). After population-wise imputation, the resulting VCF files were merged to produce one file containing all genotypes. This file was filtered to only include phenotyped lines (*n* = 5813) and SNPs genotyped in all four populations with a minor allele count (MAC) of minimum 20. This yielded a total of 12,644 SNPs for further analyses. The physical positions of all SNPs were obtained by aligning them to the Morex v3 reference genome (Mascher et al. 2021).

The SNP density plot was made using the R-package “CMplot” (Yin et al. 2021). The median number of SNPs per million base pair (Mbp) was calculated using VCFtools v.0.1.16 (Danecek et al. 2011). Finally, the median distance between neighbouring SNPs was calculated using a custom R-script.

### Population structure and outlier removal

To examine the overall population structure and to detect extreme population outliers, PCA was performed in PLINK v.1.9 (Purcell et al. 2007). Extreme population outliers were defined as genotypes where principal components 1 to 3 were closer to the median value of another population than the assigned. In total this yielded 8 outliers; 3 from 6RS, 3 from 2RS and 2 from 2RW, that were removed from the data. In addition, the genomic relationship and genetic redundancy within populations were assessed using the off-diagonal elements of the genomic relation matrix (G) as shown in Fig. S1. G was calculated by VanRaden method 1 as described in VanRaden (2008).

Genetic structure and admixture were further analysed with the software ADMIXTURE setting *K* from 2 to 40 (Alexander et al. 2009). To find the optimal value of *K*, the software-integrated CV function was used to perform a 10-fold CV at each *K* value.

### Linkage disequilibrium

Intra-chromosomal LD was estimated between all pairs of SNPs within populations. Two measurements of LD were calculated—one estimated the traditional squared allele-frequency correlations (*r*^2^) and the other corrected for kinship relationships (*r*_*V*_^2^) using the method proposed by Mangin et al. (2012). LD decay was examined by ordering SNP pairs by distance followed by binning of data into groups according to their physical distance. Bins consisted of 10 kilobase pair (kbp) intervals, i.e. the first bin contained all SNP pairs with a distance of 0–10 kbp, the next bin all pairs with a distance of 10–20 kbp etc. For each bin the average *r*^2^ or *r*_*V*_^2^ value was plotted against the average distance between marker pairs and a smooth curve was added in R using the *loess* function with a span of 0.3.

The persistence of allele phases between populations over distance were examined by considering the described SNP bins. Using the same approach as Schopp et al. (2017), linkage phase similarity (LPS) was calculated within each bin by computing the cosine similarity of *r*_*v*_ values between population pairs. The results were plotted similarly to the LD decay plots of single populations.

### Variance components and heritability estimation

Traits were analysed by fitting the following linear mixed model using the software package DMU (Madsen and Jensen 2013):

1 $${\boldsymbol{y}}\,={{\mathbf{X}}}_{\mathbf{1}}\mu +\,{{\mathbf{X}}}_{\mathbf{2}}{\mathbf{l}}+{{\mathbf{Z}}}_{\mathbf{1}}{{\mathbf{g}}}_{{\rm{a}}}+{{\mathbf{Z}}}_{\mathbf{2}}{{\mathbf{g}}}_{{\mathbf{l}}}+{{\mathbf{Z}}}_{\mathbf{3}}{\mathbf{w}}\,+\mathop{\sum }\limits_{j=1}^{15}{{\mathbf{Z}}}_{\boldsymbol{j}}{\mathbf{s}}+{\mathbf{e}}$$

Where **y** is the vector of phenotypic observations; *µ* is the overall mean; **l** is the vector of the fixed effects associated with year x location effects; **g**_a_ is the vector of genomic breeding values for lines with ${{\bf{g}}}_{{\rm{a}}}\sim N({\bf{0}},{\bf{G}}{\sigma }_{{ga}}^{2})$ where ${\sigma }_{{ga}}^{2}$ denotes the additive genetic variance and **G** is the genomic relationship matrix; **g**_l_ is the vector of residual line effects (not captured by the additive marker effects); **w** denotes the interaction between environment and genotype (year x location x line) with ${\bf{w}}\sim N({\bf{0}},{\bf{I}}{\sigma }_{w}^{2})$; *s* is a vector of spatial effects with ${\bf{s}}\sim N({\bf{0}},{\bf{I}}{\sigma }_{s}^{2})$, which captures a moving window containing the plot itself and the 14 surrounding plots within trials when analysing the 6RW, 2RW and 6RS populations (Fig. S2). In the 2RS population, no *y*-coordinates were provided, and therefore spatial effects were modelled as independent fixed effects of *x*-coordinates. **e** is a vector of residual effects with ${\bf{e}}\sim N({\bf{0}},{\bf{I}}{\sigma }_{e}^{2})$. **X**_**1**_ and **X**_**2**_ are the design matrices for the fixed effects; *µ* and **l**, respectively. **Z**_**1**_, **Z**_**2**_, **Z**_**3**_ and **Z**_**j**_ are the design matrices for the random effects; **g**_*a*_, **g**_*l*_, **w**, and **s**, respectively.

To account for inbreeding, the reported additive genetic variance component (${\sigma }_{{ga}}^{2}$) and its standard error (SE) have been multiplied by the average diagonal of the relevant **G** matrix. When the spatial variance was calculated as a moving window summing over 15 plots, the spatial variance component (${\sigma }_{{gs}}^{2}$) and its SE were reported as the estimated value multiplied by 15 to account for the contribution of 15 plots within the window.

The broad sense heritability (*H*^*2*^) and narrow sense heritability (*h*^*2*^) were calculated as follows:

2 $${H}^{2}=\,\frac{\widehat{{\sigma }_{{ga}}^{2}}+\widehat{{\sigma }_{{gl}}^{2}}}{\widehat{{\sigma }_{p}^{2}}}$$

3 $${h}^{2}=\,\frac{\widehat{{\sigma }_{{ga}}^{2}}}{\widehat{{\sigma }_{p}^{2}}}$$

Both heritability measurements were calculated on a single plot as well as a line mean (entry) level. For the single plot level, phenotypic variance was calculated as:

4 $$\widehat{{\sigma }_{p}^{2}}=\widehat{{\sigma }_{{ga}}^{2}}+\widehat{{\sigma }_{{gl}}^{2}}+\widehat{{\sigma }_{w}^{2}}+\widehat{{\sigma }_{s}^{2}}+\widehat{{\sigma }_{e}^{2}}$$

For the entry level, phenotypic variance was calculated as:

5 $$\widehat{{\sigma }_{p}^{2}}=\widehat{{\,\sigma }_{{ga}}^{2}}+\widehat{{\sigma }_{{gl}}^{2}}+\frac{\widehat{{\sigma }_{w}^{2}}}{{n}_{e}}+\frac{\widehat{{\sigma }_{s}^{2}}}{{n}_{r}}+\frac{\widehat{{\sigma }_{e}^{2}}}{{n}_{r}}$$

Where *n*_*e*_ *and n*_*r*_ indicate the number of environments and the average number of replicates per line across all environments, respectively.

### GWAS models

All GWAS analyses were performed using the software DMU (Madsen and Jensen 2013). GWAS was performed on a set of SNPs with MAC ≥ 30. When combining populations, SNPs with a total MAC < 30 or with a MAC < 10 in either population were excluded. GWAS was done as a single SNP regression using an expanded version of the model stated in Eq. 1. It was performed within populations (single-population GWAS) and by combining populations (multi-population GWAS) using either a univariate model (MP1) or a multivariate model combining populations (MP2). In the MP1 model, the GWAS trait is treated as the same across populations. The model is an expansion of the single-population model and includes a fixed population effect. Further, the model nests fixed effects of environments (**l**, Eq. 1) within populations. In contrast, MP2 treats the same trait in different populations as genetically correlated using a multivariate version of the previously presented GWAS models. In addition to the estimation variance of SNP effect estimates, the covariance between estimation errors from SNP regression was estimated for each pair of populations in MP2.

### GWAS significance test and correction for multiple testing

Significance of GWAS marker effects were assessed using *p* values calculated by applying a Wald test to *z* scores:

6 $${z}_{i}=\frac{\widehat{{b}_{i}}}{{SE}(\widehat{{b}_{i}})}$$

Using the single-population and MP1 models, ${\widehat{{b}_{i}}}$ is the estimated regression coefficient of SNP *i*. When applying the test to MP2, ${\widehat{{b}_{i}}}$ is the sum of the *n*_*p*_ scaled estimated regression coefficients when combining *n*_*p*_ populations. Thus in MP2 ${SE}({\widehat{{b}_{i}}})$ the following equations apply:

7 $${b}_{{scaled},i}=\mathop{\sum }\limits_{j=1}^{{n}_{p}}\frac{\widehat{{b}_{i,j}}}{{SE}(\widehat{{b}_{i,j}})}$$

${\widehat{{b}_{i,{j}}}}$ refers to the estimated regression coefficient of SNP *i* in population *j*.

In order to calculate the *z* scores of SNP regressions from MP2, the SE of the estimated SNP effects are calculated as follows:

8 $${SE}\left({\widehat{b}}_{{scaled},i}\right)=\sqrt{{{\bf{a}}}^{{\bf{T}}}{{\bf{V}}}_{{\bf{i}}}{\bf{a}}}$$

Where **a** is a column vector of ones with dimension 1 × *n*_*p*_ where *n*_*p*_ is the number of included populations. **V**_i_ denotes the variance-covariance matrix of the scaled regressions for SNP *i* and has the dimension of *n*_*p*_ × *n*_*p*_. Due to the scaled nature of the SNP regressions, all the diagonal elements in **V** are one. Each covariance between SNP regressions in different populations were estimated as follows:

9 $${\mathrm{cov}}({\widehat{b}}_{{scaled}\,i,j},{\widehat{b}}_{{scaled}\,i,k})\,=\,\frac{{\mathrm{cov}}({\widehat{{b}_{i,j}}},\,{\widehat{{b}_{i,k}}})}{{SE}({\widehat{{b}_{i,j}}}){SE}({\widehat{{b}_{i,k}}})}$$

Where ${\widehat{b}}_{{scaled\; i},j}$ and ${\widehat{b}}_{{scaled\; i},k}$ are the scaled regressions of the *i*th SNP in populations *j* and *k*, respectively.

To correct for multiple testing a Bonferroni correction was applied setting the genome-wide significance threshold to 0.05/12,644 = 3.95E-06.

### Further statical analyses and modelling

The theoretical proportion of additive genetic variance explained (PVE) by the *i*th marker was calculated as follows:

10 $${{PVE}}_{i}\,=\,\frac{{2* p}_{i}* (1-{p}_{i})* {\widehat{{b}_{i}}}^{2}}{\widehat{{\sigma }_{g\alpha }^{2}}}$$

Where *p*_*i*_ and ${b}_{i}$ are the allele frequency and the estimated regression coefficient of the *i*th marker, and $\widehat{{\sigma }_{g\alpha }^{2}}$ (not to be confused with $\widehat{{\sigma }_{{ga}}^{2}}$) is the additive genetic variance before multiplication with the average diagonal elements of the **G** matrix.

Prior to modelling the genetic correlation between heading date and lodging within a population, the data was limited to observations of plots scored for both traits. The genetic correlation was then modelled by expanding Eq. 1 to a bivariate model which included residual covariance.

### Power calculations

Power analyses were performed for each GWAS by iterating over values of SNP effects (*b*) ranging from 0 to $\sqrt{2}$ by steps of 0.01. For each *b* value, the power calculations were performed for a set of non-centrality parameter (NCP) values as described by Sham and Purcell (2014):

11 $${\rm{NCP}}={\left(\frac{{\rm{b}}}{{\rm{SE}}({\widehat{{\rm{b}}}})}\right)}^{2}$$

The SE of each SNP effect (${\rm{SE}}({\widehat{{\rm{b}}}})$) originated from our actual GWAS on each trait, which was reduced to the common set of 4812 markers with a MAC ≥ 30 in all populations.

In the single-population and MP1 models, NCPs were obtained by dividing the given *b* value by the vector of ${SE}(\hat{b})$ produced by the GWAS models. For MP2, NCP ratios were obtained by dividing the corresponding scaled *b* value (Eq. 7) by the vector of SE of scaled *b* values (Eq. 8). The NCPs were used as test statistics in a non-central chi-squared test with 1 degree of freedom and a type 1 error rate set to the overall Bonferroni significance threshold. As power calculations were applied to each NCP value, we averaged over SNPs to obtain one power measurement per value of *b*^*2*^.

## Results

### Distribution of SNP markers

**Fig. 1:**
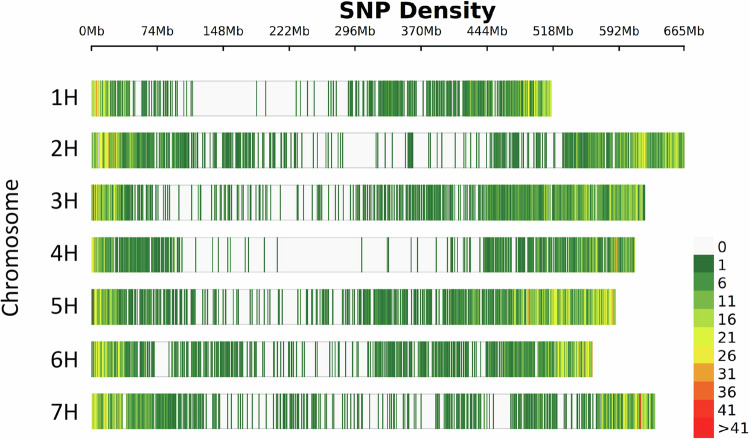
Density plot of the 12,644 SNP markers. Distribution of SNPs across the seven barley chromosomes. The colours of the bars refer to SNP count within a 1 Mbp window.

A total of 5805 barley accessions were genotyped using a set of 12,644 high-quality SNPs. The largest number of SNPs were mapped to chromosome 5H (2396) whereas chromosome 4H contained the smallest number of SNPs (1258). Relatively few markers were located in the centromeric regions of chromosomes, the majority were located in non-centromeric regions (Fig. 1). With this unequal distribution in mind, we reported SNP statistics based on medians instead of means. The median distance between neighbouring SNPs was 38.9 kbp, while the number of SNPs per 1 Mbp ranged from 0 to 45 (Table S3).

### Population structure and LD decay

**Fig. 2:**
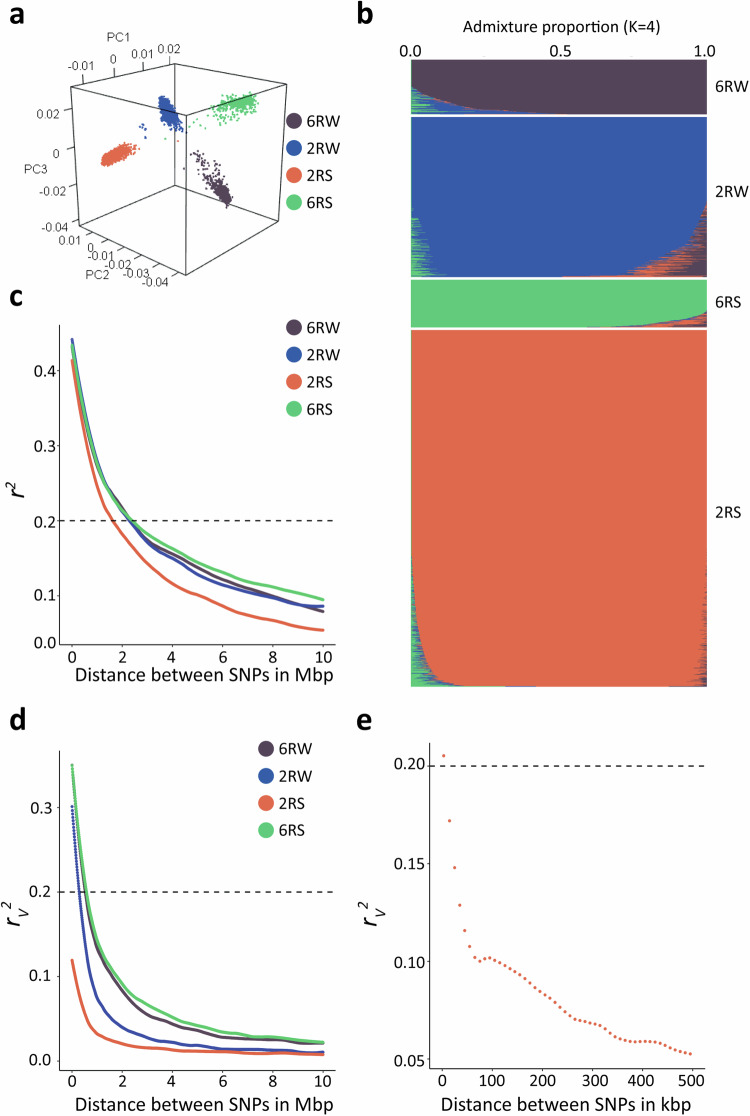
Population structure and LD of the four breeding populations. **a** the first three principal components displaying the genetic structure of the four breeding populations. A rotating version of the plot can be accessed at: https://figshare.com/s/d399272943cb2384ce46. **b** A barplot displaying the genetic admixture proportions of individuals at *K* = 4. **c**, **d** LD decay of populations without (**c**) and with correction for kinship (**d**). The horizontal lines display *r*^*2*^ or *r*_*v*_^*2*^ at 0.2. **e** Zoomed in version of population-corrected LD (*r*_*v*_^*2*^) for the 2RS population at SNPs distanced up to 500 kilobase pairs apart. In all panels, different breeding populations are colour-coded.

To examine the genetic relationships between the four breeding populations we carried out PCA based on genotypic information from all SNP markers. The first three principal components (PCs) collectively explained 38% of across population genotypic variance and clearly separated the four breeding populations. PC1, which explained 23% of the total SNP variance, separated spring-type breeding populations (2RS, 6RS) from winter types (2RW, 6RW). PC2, which explained 9% of the variance, caused a clear distinction of row-types. PC3 gave rise to a further genetic separation of 6RW and the remaining populations (Fig. 2a). For the admixture analysis, the CV error was minimized at the maximum tested value of *K* = 40. The high value of *K* was caused by comprehensive family structure in the data causing difficulties in determining the optimal number of ancestral populations. Nevertheless, results at *K* = 4 clearly distinguished the four breeding populations, which generally showed little admixture (Fig. S3, Fig. 2b). To investigate how the LD differed between the populations, the average correlation between SNP pairs, before (*r*^*2*^) and after correction for kinship (*r*_*v*_^*2*^), were plotted as a function of physical distance. The LD decay was estimated as the distance where LD drops to 0.2. The LD patterns were similar for 6RS (2.4 Mbp), 6RW (2.3 Mbp) and 2RW (2.3 Mbp), but decayed faster in the 2RS population (1.6 Mbp) (Fig. 2c). As expected, much of the observed LD was due to extensive family structure and kinship rather than physical linkage. Consequently, LD decreased rapidly when correction for kinship was done. The physical linkage between markers extended 285 kbp in the 2RW population, 575 kbp in the 6RW population and 625 kbp in the 6RS population. In the 2RS population, correction for kinship resulted in the LD decaying so rapidly that on average LD was below 0.2 after 3016 bp (Fig. 2d, e).

### Trait variances and heritability

All populations were scored for heading date and lodging (Fig. S4). In general, the 6RW population was scored in fewer environments (6–9) than the remaining populations, which were all scored in 9–18 combinations of year and location. Winter types showed earlier heading dates than spring types, as expected due to their earlier sowing, whereas populations with different growth and/or row types behaved similar for lodging (Table 2).

**Table 2:** Phenotypic data overview of all traits in the four populations.

Trait	Population	Min.^a^	Max.^b^	Mean	SD^c^	CV^d^	n.o.^e^	n.e. ^f^
Heading date	6RW	3.0	25.0	13.9	4.1	0.29	3681	9 (4, 5)
	2RW	1.0	40.0	14.6	6.1	0.42	9712	18 (7, 5)
	6RS	32.0	73.0	53.6^g^	9.3	0.17	4911	18 (4, 5)
	2RS	32.0	66.0	47.0	7.6	0.16	13.374	18 (9, 7)
Lodging	6RW	1.0	8.0	2.0	1.7	0.85	1624	6 (3, 4)
	2RW	1.0	9.0	2.1	2.0	0.95	8899	16 (7, 6)
	6RS	0.9	8.0	1.9	1.3	0.68	2405	9 (4, 5)
	2RS	1.0	9.0	2.8	2.0	0.71	17.362	15 (8, 5)

^a^Min., Minimum trait observation.

^b^Max., Maximum trait observation.

^c^SD, Standard deviation.

^d^CV, Coefficient of variation.

^e^n.o., number of observations.

^f^n.e., number of unique year x location combinations (environments)—listed in parenthesis is the number of years followed by the number of locations.

^g^The later heading date of the 6RS population compared to the 2RS is due to later sowing of the 6RS population. This is the case as the populations are grown in different geographic regions. If the populations were grown in the same location, the 6RS heading date is expected to be 10–14 days earlier than 2RS.

For all populations, the heritability for heading date was greater than for lodging. The broad sense entry-mean heritability ranged from 0.93 (6RW, 6RS) to 0.95 (2RS) for heading date and from 0.52 (6RW) to 0.80 (2RS) for lodging. The phenotypic variance was partitioned into five components: Additive genetics captured by SNP markers (σ_ga_^2^), the remaining line effect (σ_gl_^2^), spatial effects (σ_s_^2^), interaction between lines and environments (σ_w_^2^) and error (σ_e_^2^). For heading date, the largest proportion of variance was assigned to the additive genetic variance in all populations. The remaining line effect, i.e. genetic variance that is either non-additive or additive effects not captured by the SNPs, was negligible for the 2RW and 6RS populations, but contributed 3–7% of the overall trait variance in 6RW and 2RS. The relative variance explained by GxE was large in the 2RW population where it explained 29% of the observed variance. The same parameter only explained 16–19% of the overall variance in the remaining populations. Spatial effects accounted for up to 12% of the overall variance.

For lodging the largest proportion of variance was explained by GxE or the model’s error term. The relative variance proportion explained by GxE varied from 21% (6RS) to 40% (2RW), whereas the additive genetic component only explained 17–33% of the phenotypic variance. The relative contribution of spatial effects to lodging variation was between 10% and 15% for all populations except the 2RS population where spatial effects only contributed 2% to the overall variation. Genetic effects not captured by the additive term explained none of the observed variance in the populations except for 2RS where it explained 6% (Table 3).

**Table 3:** Trait variances and heritability in the four populations.

		Population			
Trait	Parameter	6RW	2RW	6RS	2RS
Heading date	σ_ga_^2^	3.80 (0.38)	6.74 (0.50)	2.58 (0.24)	2.93 (0.18)
	σ_gl_^2^	0.21 (0.09)	7.11E- 03 (8.11E- 04)	4.13E -06 (0.06)	0.32 (0.04)
	σ_w_^2^	1.20 (0.08)	3.51 (0.12)	0.82 (0.05)	0.74 (0.04)
	σ_s_^2^	0.77 (0.04)	0.27 (3.5E- 02)	0.52 (0.04)	0.24 (0.04)
	σ_e_^2^	0.49 (0.02)	1.78 (0.04)	0.98 (0.03)	0.50 (0.01)
	*H*^*2*^ (entry)	0.93 (8.44 E- 03)	0.94 (5.98E-03)	0.93 (7.37E-03)	0.95 (3.71 E-03)
	*h*^*2*^ (entry)	0.88 (0.03)	0.94 (0.01)	0.93 (0.03)	0.86 (0.02)
	*H*^*2*^ (plot)	0.62 (0.03)	0.55 (0.03)	0.53 (0.03)	0.69 (0.02)
	*h*^*2*^ (plot)	0.59 (0.04)	0.55 (0.03)	0.53 (0.04)	0.62 (0.02)
Lodging	σ_ga_^2^	0.46 (0.08)	0.35 (0.04)	0.38 (0.05)	0.79 (0.07)
	σ_gl_^2^	2.37E- 06 (0.08)	8.4E -03 (0.03)	5.4E -07 (0.03)	0.21 (0.03)
	σ_w_^2^	0.79 (0.09)	0.85 (0.03)	0.24 (0.03)	1.06 (0.04)
	σ_s_^2^	0.34 (1.38E -2)	0.32 (5.03 E -03)	0.11 (1.88E -2)	0.08 (0.08)
	σ_e_^2^	0.65 (0.04)	0.58 (0.02)	0.42 (0.02)	1.10 (0.02)
	*H*^*2*^ (entry)	0.52 (0.07)	0.66 (0.03)	0.78 (0.03)	0.80 (0.01)
	*h*^*2*^ (entry)	0.52 (0.08)	0.65 (0.05)	0.78 (0.07)	0.63 (0.04)
	*H*^*2*^ (plot)	0.21 (0.05)	0.17 (0.02)	0.33 (0.04)	0.31 (0.02)
	*h*^*2*^ (plot)	0.21 (0.04)	0.17 (0.02)	0.33 (0.05)	0.24 (0.02)

Listed in parantheses are the standard errors of the parameter estimate.

### Single-population GWAS

**Fig. 3:**
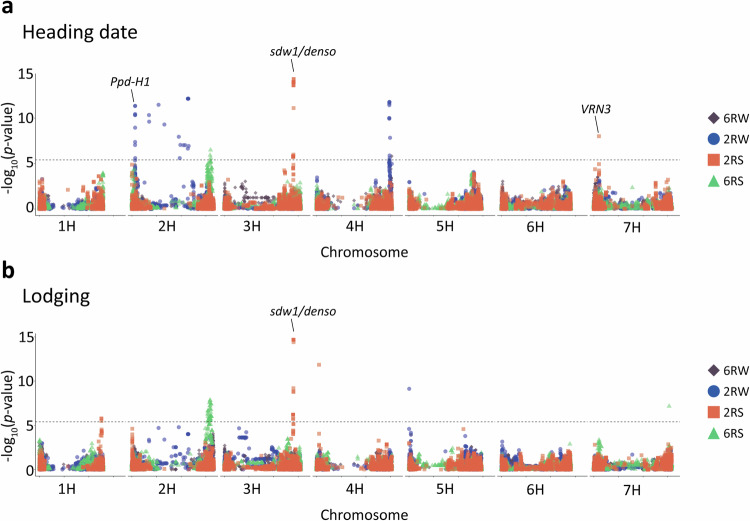
Manhattan plots for single-population GWAS. Overlaid Manhattan plots of single-population GWAS results for (**a**) heading date and (**b**) lodging. The *y*-axes display −log_10_ of *p*-values. The results for each population are displayed with a unique colour and shape. The *x*-axes display the genomic position of markers by chromosome. The horizontal dashed lines indicate the Bonferroni-corrected significance threshold at −log_10_(*p*) = 5.4. The position of the well-known genes *Ppd-H1, sdw1/denso,* and *VRN3* are indicated with labels.

We performed single-population GWAS and identified genomic regions associated with heading date and lodging within populations (Fig. 3, Figs. S5–6). We detected 37, 23, and 3 significant marker-trait associations (MTA) for heading date in the 2RW, 2RS and 6RS population, respectively. For the 6RW population, we found only one significant MTA. The identified MTAs were located on chromosomes 2H, 3H, 4H and 7H (Fig. 3a).

Single-population GWAS for lodging revealed 19, 32, and 1 MTAs in 2RS, 6RS, and 2RW, respectively. No significant MTAs were detected for lodging in the 6RW population. The lodging MTAs were spread across all chromosomes except 6H (Fig. 3b). A comprehensive list of all significant MTAs for both traits can be found in Table S4. To point towards candidate genes, MTAs were grouped into candidate QTLs by considering the full genomic interval spanned by the significant markers contributing a visual GWAS peak. In Table 4 each of the resulting candidate QTLs are described by the most significant SNP and its closest gene. In total, single-population analyses pointed to seven QTLs for heading date and six QTLs for lodging. Among the candidate QTLs for heading, one detected in 2RS was located in a major vernalization gene *VRN-3*, and another detected in 2RW was located in the photoperiod gene *Ppd-H1* (Turner et al. 2005; Yan et al. 2006) (Fig. 3a).

**Table 4:** Single-population GWAS candidate QTLs.

Trait	Pop	Region^a^	Lead SNP	MAC^b^	*p*	Gene/protein function^c^	PVE(%)^d^			
							6RW	2RW	6RS	2RS
Heading date	2RW	2H:25,680,956-26,781,024 bp	Marker6863	1101	4.57E-12	*Ppd-H1* /Pseudo-response regulator 7	4.6	16.6	6.9	<0.1
	2RW	2H:138,380,194-460,538,308 bp	Marker5780	68	7.32E-13	Histidinol dehydrogenase, chloroplastic	0.2	7.0	1.1	<0.1
	6RS	2H:634,395,585-639,427,862 bp	Marker3101	81	3.21E-07	NA	<0.1	<0.1	9.7	<0.1
	2RS	3H:558,347,932-562,968,862 bp	Marker11212	31	5.14E-15	NA	<0.1	<0.1	1.3	3.5
	2RW	4H:589,422,386-595,541,830 bp	Marker4161	225	1.68E-12	NA	NA	7.0	<0.1	<0.1
	6RW	4H:609,359,514 bp	Marker1077	393	1.54E-06	NA	6.9	1.4	0.1	NA
	2RS	7H:41,824,853 bp	Marker33	34	9.86E-09	*VRN3*	0.2	<0.1	2.1	1.0
Lodging	2RS	1H:501,998,439-502,124,385 bp	Marker11732	60	1.68E-06	NAD-dependent epimerase/dehydratase domain-containing protein	0.8	<0.1	0.5	1.8
	6RS	2H:616,147,288-644,960,866 bp	Marker7239	59	1.47E-08	HvCP3-31/Cysteine protease	1.9	3.0	19.5	0.3
	2RS	3H:558,347,932-562,272,693 bp	Marker11289	30	2.13E-15	Oxidoreductase	0.3	1.3	1.8	8.8
	2RS	4H:19,092,878 bp	Marker8874	365	1.43E-12	NA	3.1	NA	0.2	4.7
	2RW	5H:1,331,317 bp	Marker2721	609	7.34E-10	NA	0.4	9.5	0.2	0.6
	6RS	7H:611,450,495 bp	Marker4844	32	6.85E-08	NA	3.3	<0.1	12.0	<0.1

NA results from the SNP marker being monomorphic in the population.

*Pop* populations, *NA* not applicable.

^a^The associated region is defined as the interval covered by the GWAS peak, i.e. the start position corresponding to the associated SNP closest to the 5’ end of the chromosome and the end position corresponding to the associated SNP closest to the 3’ end of chromosome.

^b^Minor allele count in the analysed population.

^c^For intergenic SNP markers, the closest gene is reported.

^d^Population-wise percentage of additive genetic variance explained by the lead SNP marker when using the estimated SNP regressions from single-population GWAS model on 6RW, 2RW, 6RS or 2RS.

Further, single-population GWAS found two QTLs associated with both traits, which are results of the broad 6RS-specific candidate QTL on chromosome 2H and the 2RS-specific candidate QTL on chromosome 3H. The latter colocalizes with the well-known semi-dwarfing gene *sdw1/denso* (Jia et al. 2009). To explain the overlap of traits in the two populations, we found significant genetic correlations between heading date and in the 6RS (−0.51) and 2RS (−0.31) populations. In contrast, genetic correlations between the traits were non-significant in the 6RW population and positive (+0.33) in the 2RW population (Table S5).

We observed no physical overlap of QTLs between any of the populations within traits. Most of the identified QTLs were found in the populations with larger sample size (2RS and 2RW). Notably, the 2RW population yielded many MTAs for heading date spread across chromosome 2H making it difficult to separate and identify QTLs.

### Persistence of allele phases between populations

**Fig. 4:**
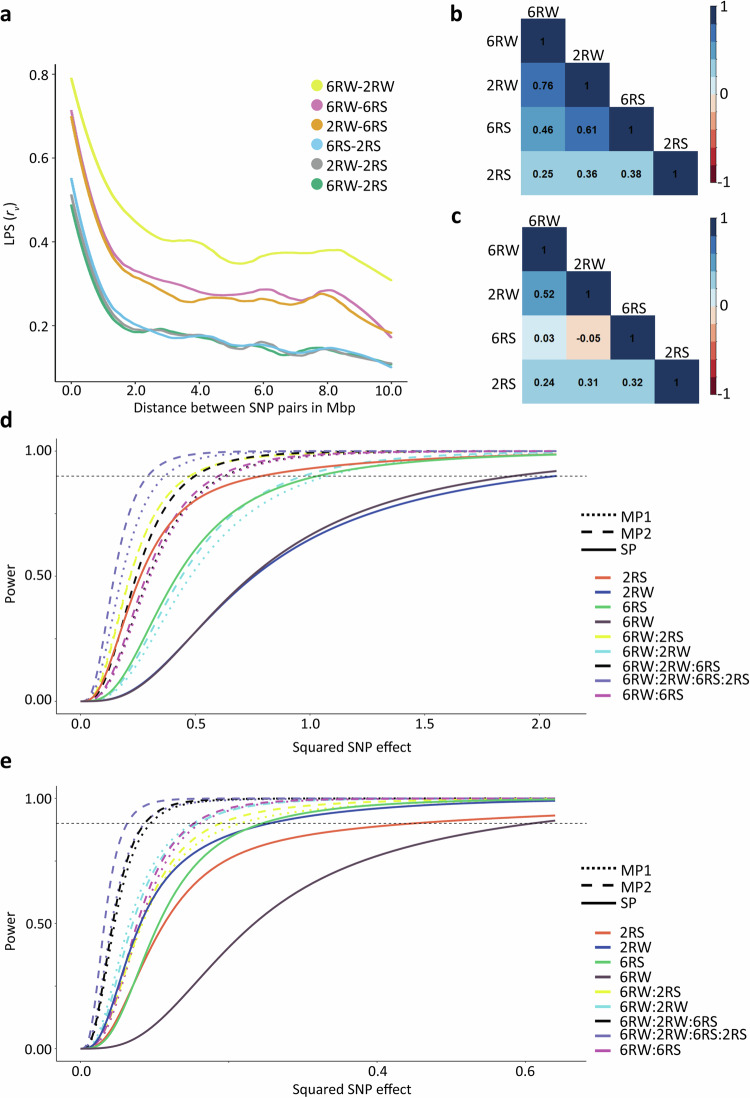
The basis and potential for combining populations in GWAS. **a** Linkage phase similarity (LPS) between pairwise populations for SNP pairs at different distances. SNP pairs are binned in groups of 10,000 bp intervals. **b** Genetic correlations of heading date between populations. **c** Genetic correlations of lodging between populations. **d**, **e** Power plots for different GWAS models applied to heading date (**d**) and lodging (**e**). The *x*-axes show the squared value of a hypothetic SNP effect. The *y*-axes show the statistical power of GWAS for a given marker size. Different lines types are used to display different models, whereas line colours refer to the combination of populations subjected to GWAS. The dashed horizontal lines indicate a power of 0.9.

For both traits, only one marker exhibited significance above the threshold in the 6RW population. To gain more statistical power to uncover MTAs in this population, we sought to transfer information from the other populations. To access how well-suited the different breeding programs were for combined GWAS, we studied LD phasing between the populations. For all pairwise populations, the LPS decreased with increasing marker distance. For markers distanced <10 kbp apart, phases tended to be preserved between populations. The largest LPS (0.79) was observed between 6RW and 2RW at a distance up to 14 kbp, and the persistence of allele phases for these two populations remained high (LPS > = 0.5) for markers distanced up to 1.4 Mbp apart. We also observed moderate agreement between the linkage phases of 6RW and 6RS, and between 2RW and 6RS where the LPS at short distances (<10 kbp) were up to 0.70–0.71 and remained high (LPS > = 0.5) up to distances of 595–645 kbp. In contrast, the 2RS population showed a lower consistency in linkage phases with the other populations. The lowest LPS was observed for 6RW and 2RS where markers distanced within 10 kbp showed an LPS of 0.49 (Fig. 4a). In general, we found that the persistence of phases highly reflected the genetic relationship between populations (Fig. 2a, b).

As the persistence of phases remained positive for all pairwise comparisons of populations, it suggested that on average we did not observe reverse phases between populations. However, it was still possible that some regions had reverse phases between populations. To evaluate this, we studied the LPS between correlations of all possible marker pairs located within overlapping windows of 1 Mbp. We only observed negative LPS values of windows three times across all chromosomes and pairwise comparisons of populations. The most negative of these (LPS = −0.27) was found by comparing 6RS and 2RS on chromosome 1H (Tables S6–S11). Based on this, we concluded that for the presented data, phase inversions between populations were not common, hence we did not expect SNP effects in different populations to cancel each other out when applying multi-population GWAS models.

### Cross-population genetic correlations and multi-population GWAS

Despite finding no overlapping MTAs in the four populations for the same trait, a trait scored in different populations could still share some of their genetic architecture i.e. similar QTL effects across genetic backgrounds. Using a bivariate GWAS model we estimated the cross-population genetic correlations. We observed higher cross-population genetic correlations for heading date compared to lodging. For heading date, the correlation between populations decreased with increasing genetic distance resulting in the largest genetic correlation (+0.76) between 6RW and 2RW, and the smallest genetic correlation (+0.25) between 6RW and 2RS (Fig. 4b). For lodging we observed the largest genetic correlation (+0.52) between 6RW and 2RW, and the smallest genetic correlations between 6RW and 6RS (+0.03) and 2RW and 6RS (−0.05) (Fig. 4c).

We combined the 6RW data with datasets from other populations in multi-population GWAS analyses using two different models. MP1 combined the populations in a univariate model and thereby assumed a cross-population genetic correlation of +1. To account for the inaccuracy of this assumption, we applied MP2, which was a multivariate model treating a trait scored in the different populations as different but genetically correlated traits. Since our aim was to improve GWAS of the 6RW population, we combined it with one of the other populations at a time (6RW:2RW, 6RW:6RS, 6RW:2RS) and by using data from the two genetically closest related populations (6RW:2RW:6RS), or all four populations (6RW:2RW:6RS:2RS).

Power analyses revealed that the multi-population GWAS models yielded higher power to detect MTAs compared to the single-population GWAS model under the assumption that the QTL effects were the same across populations. For heading date, MP2 obtained a power of 90% for SNP effects as low as 0.3 days per alternative allele when combining all four populations, whereas effect sizes needed to be 1.4 days per alternative allele for similar power in single-population GWAS in 6RW. For lodging in 6RW, an effect size as low as 0.07 per alternative allele resulted in a theoretical power of 90% when applying MP2 GWAS to all four populations, whereas effect size per alternative allele needed to be much larger in the 6RW population (*b* = 0.8) to obtain the same power using the single-population models (Fig. 4d, e).

**Fig. 5:**
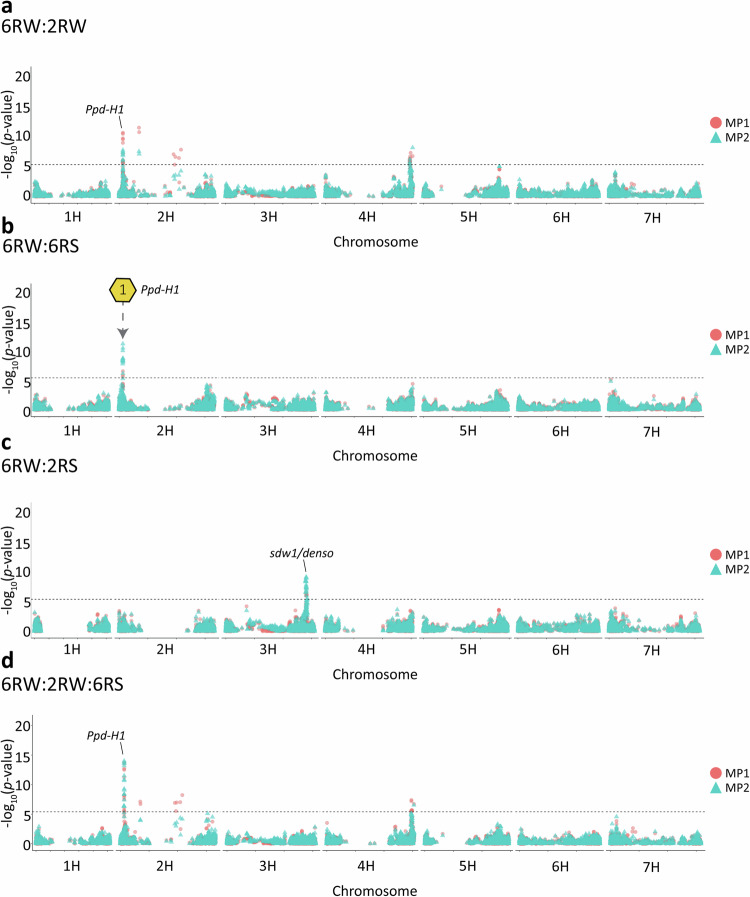
Overlaid Manhattan plot for heading date using multi-population GWAS models. The red circles display the GWAS results from the univariate model (MP1) and the blue triangles display the results from the multivariate model (MP2) when performing GWAS on:. **a** 6RW:2RW. **b** 6RW:6RS. **c** 6RW:2RS. **d** 6RW:2RW:6RS. The horizontal dashed lines indicate the Bonferroni-corrected genome-wide significance threshold at −log_10_(*p*) = 5.4. Hexagons with numbers point towards candidate QTLs that were not found when performing single-population GWAS on the involved populations. Golden hexagons refer to high-confidence candidate QTLs found by the MP2 model alone or by both models. Candidate QTL1 points to chromosome 2H at position 24,418,356–25,879,587 bp. The position of the well-known genes *Ppd-H1 and sdw1/denso* are indicated with labels.

**Fig. 6:**
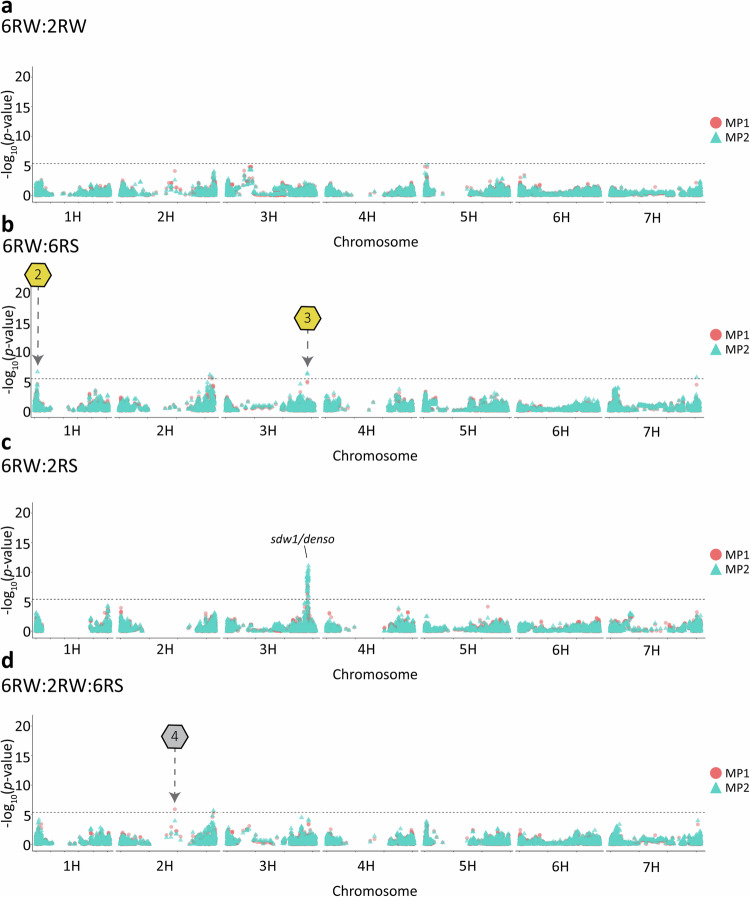
Overlaid Manhattan plot for lodging using multi-population GWAS models. The red circles display the GWAS results from the univariate model (MP1) and the blue triangles display the results from the multivariate model (MP2). **a** 6RW:2RW. **b** 6RW:6RS. **c** 6RW:2RS. **d** 6RW:2RW:6RS. The horizontal dashed lines indicate the Bonferroni-corrected genome-wide significance threshold at −log_10_(*p*) = 5.4. Hexagons with numbers point towards candidate QTLs that were not found when performing single-population GWAS on the involved populations. Golden hexagons refer to high-confidence candidate QTLs found by the MP2 model alone or by both models. Candidate QTL 2 points to chromosome 1H at position 19,595,378 bp; Candidate QTL 3 points to chromosome 3H at position 570,634,774–570,635,897 bp; Candidate QTL 4 points to chromosome 2H at position 380,356,960 bp.

In multi-population GWAS, combining the target population (6RW) with other breeding populations gave rise to a total of 15 and 9 candidate QTLs for heading date and lodging, respectively (Figs. 5, 6, S5–6). No additional candidate QTLs were detected when combining all four populations (Fig. S7). We found that the SNP effects in MP1 was significantly dominated by the larger population and substantially overestimated (Table S12). As a result, we do not consider MP1-specific QTLs as valid candidates, and therefore, Table 5 only reports the candidate QTLs identified by MP2. Since some of the same candidate QTLs within traits were found across combinations of populations, the detected candidate QTLs can be reduced to four and five non-overlapping candidate QTLs for heading date and lodging, respectively (Table 5). Among the MP2 multi-population analyses, we identified three candidate QTLs that were only detected when combining populations and not when subjecting the included populations to single-population GWAS (Figs. 5, 6). The locations of these QTLs are highlighted in Figs. 5 and 6. Notably, when combining 6RW and 6RS in GWAS for heading date, associations located on chromosome 2H 24.4–25.9 Mbp appear. This region overlaps with the *Ppd-H1* gene detected for heading date in the 2RW population (Figs. 3a, 5b). The proportion of genetic variance explained by all MP2 GWAS QTLs ranged from 0.1% to 13.8% in 6RW, from 0.1% to 15.1% in 2RW, from 0.2% to 19.4% in 6RS, and from 0.5% to 11.2% in 2RS. Of the identified QTLs for heading date, the chromosome 2H signal located at 24.4–25.9 Mbp explained the largest proportion of genetic variance in 6RW (8.3%) (Table 5). For the lead marker in this region, 6RW homozygotes for the G allele had on average 14.7 days from May 1st until heading, whereas homozygotes for the T allele had only 13.4 days. In the remaining populations, substituting homozygosity of the G allele with homozygosity of the T allele, reduced the average heading date from 17.2 to 13.4 days after May 1st (2RW), and from 53.7 to 48.4 days after May 1st (6RS). No variation was observed for the marker in the 2RS population (Fig. S8). Among the identified candidate QTLs for lodging, the lead marker on chromosome 2H at 650.5 Mbp explained the largest amount of genetic variation in 6RW (13.8%) (Table 5). Homozygotes for favourable allele (G/G) had a mean score of 1.8 across all environments, whereas homozygotes for the unfavourable allele (A/A) had a mean score of 3.2. In the remaining populations, the average lodging score of genotypes changed from 3.1 to 2.1 (2RW), from 3.0 to 1.9 (6RS), and from 4.5 (2RS) to 2.8 when substituting A/A with G/G (Fig. S9).

**Table 5:** Multi-population (MP2) GWAS candidate QTLs.

Trait	Pop	Region^a^	Lead SNP	*p*	Gene / protein function	PVE(%)^b^			
						6RW	2RW	6RS	2RS
Heading date	6RW:2RW	2H:25,802,784-26,109,060 bp	Marker6863	1.44E-08	*Ppd-H1*	4.3	15.1	-	-
	6RW:2RW	2H:138,380,194-139,779,245 bp	Marker2972	2.40E-08	NA	0.3	4.8	-	-
	6RW:2RW	4H:588,401,196-609,359,514 bp	Marker1077	5.47E-09	NA	7.0	1.2	-	-
	6RW:6RS	2H:24,418,356-25,879,587 bp	Marker8831	6.94E-12	NA	8.3	-	6.9	-
	6RW:2RS	3H:557,935,365-569,272,255 bp	Marker5620	6.44E-10	Oxidoreductase	0.3	-	-	3.6
	6RW:2RW:6RS	2H:24,418,356-25,936,706 bp	Marker6863	9.96E-15	*Ppd-H1*	4.1	15.1	6.6	-
	6RW:2RW:6RS	4H:609,359,514 bp	Marker1077	2.89E-07	NA	7.0	1.1	0.2	-
Lodging	6RW:6RS	1H:19,595,378 bp	Marker4096	2.67E-07	NA	8.4	-	13.8	-
	6RW:6RS	2H:634,395,585-650,512,028 bp	Marker7239	8.03E-07	HvCP3-31/Cysteine protease	1.9	-	19.4	-
	6RW:6RS	3H:570,634,774-570,635,897 bp	Marker1471	4.73E-07	NA	9.6	-	12.8	-
	6RW:6RS	7H:611,450,495 bp	Marker4844	2.38E-06	NA	3.2	-	11.9	-
	6RW:2RS	3H:557,935,365-569,419,208 bp	Marker10700	8.47E-12	DUF295 domain-containing protein	2.0	-	-	10.8
	6RW:2RW:6RS	2H:650,512,028 bp	Marker6536	1.99E-06	NA	13.8	0.7	4.6	-
	6RW:2RW:6RS:2RS	2H:643,481,329 bp	Marker5756	1.64E-06	Metal ion-binding	0.6	3.4	9.7	0.5
	6RW:2RW:6RS:2RS	3H:557,935,365-568,461,838	Marker10020	6.45E-08	NA	0.1	0.1	2.8	11.2

NA results from the SNP marker being monomorphic in the population or from unannotated genes.

*Pop* populations, *NA* not applicable.

^a^The associated region is defined as the interval covered by the peak, i.e. the start position corresponding to the associated SNP closest to the 5’ end of the chromosome and the end position corresponding to the associated SNP closest to the 3’ end of chromosome.

^b^Population-wise percentage of additive genetic variance explained by the lead SNP marker.

## Discussion

### Population structure and LD decay reflected the breeding history of barley

In agreement with previous studies, we found that breeding activities have resulted in strong population structure of barley—primarily due to the separation of winter and spring types and secondarily due to the separation of ear row number (Rostoks et al. 2006; Wang et al. 2012). Further, the observed LD decay rates i.e. the amount of experienced recombination of the different populations highly reflect the breeding history of barley. The slower LD decay of the 6-rowed populations can be explained by 6-rowed barley descending from 2-rowed barley (Backes et al. 2006; Komatsuda et al. 2007). We especially observed an extremely fast LD decay in the 2RS population. This clearly reflected that the earliest breeding programs in Europe focused on intensive breeding of 2-rowed spring barley, while the concurrent winter barley breeding programs were minor (Fischbeck 1992). We found a moderate to high LPS when comparing linked markers in the 6RW population with the 2RW and 6RS population, but a low LPS when comparing the 6RW and 2RS population. This is consistent with previous findings reporting a decreasing LPS with increasing genetic distance between barley breeding populations (Hamblin et al. 2010). In contrast to our results, Hamblin et al. (2010) reported a low consistence of phases even for close markers. Nevertheless, we found almost no cases of reverse phases. Although surprising, this might be explained by few events of recombination since the separation of the involved breeding programs. The first line breeding programs for Nordic cultivation of barley can be traced back to the beginning of the 20th century (Ortiz et al. 2002). The low haplotype divergence observed between types may be explained by the effective recombination in cultivated barley being low, as 10 years typically has passed from the first crossings to cultivar releases in barley. Further explanations can be found in introgression between types e.g. the transfer of malting qualities between populations (Stockinger 2021).

Another consequence of selective barley breeding is the scarcity of SNPs in the pericentromeric region of chromosome 2H in the 2RS population. Mascher et al. (2017) hypothesized that this might be attributed to extensive breeding efforts aimed at the favorable *HvCEN* allele found in this region. *HvCEN* contributes to both determination of growth habits and geographical adaptations, making it a likely GWAS result for heading date in the remaining populations where some genetic variation still exists in this region. In this context, we observed that the 2RW population gave rise to multiple heading date associations scattered across the pericentromeric region on chromosome 2H. As we have no markers located in *HvCEN* (the closest being 2–3 Mbp away) and a general absence of sufficient recombination in centromeric regions, the associations might report *HvCEN*.

### Lodging showed a more quantitative nature than heading date

We observed that lodging had a lower heritability than heading date. This was caused by most of the total variance in lodging being ascribed to environmental (GxE) and residual effects. The larger environmental influence was expected, as stem lodging resistance is a combination of many traits e.g. plant height, crown width, stem diameter, and the structural stem components, all interacting with environmental factors (Li et al. 2022). The larger residual variance might indicate greater measurement errors linked to the lower resolution of the scale for scoring lodging (1–9). It should be pointed out, that we observed a low degree of lodging in all populations. Interestingly, we found a lower heritability for lodging in winter populations than in spring populations. We attribute this to the larger environmental effects (GxE and spatial effects) on lodging experienced by the winter populations, which might be related to winter barley staying in the field for a prolonged period compared to spring barley. Further, we observed a negative genetic correlation between lodging and heading date in the spring populations—a trend that has previously been reported in Northern spring barley breeding material (Göransson et al. 2019). The relationship between these traits led to a common candidate QTL on chromosome 3H, observed in the GWAS on 2RS alone and in the following combinations 6RW:2RS (both traits), and 6RW:2RW:6RS:2RS (lodging only). The QTL includes the *sdw1/denso* gene, a semi-dwarfing gene where the mutant allele is widely used in European spring barley to improve lodging resistance and delay heading (Jia et al. 2009; Kuczyńska et al. 2013).

### Multi-population GWAS increased power to detect associations and MP2 had higher precision than MP1

We tested the power of the multi-population models compared to the single-population model in the theoretical case where a SNP effect is the same in all tested populations. Consistent with previous studies, combining populations resulted in higher power than performing GWAS in either of the populations alone (Gebreyesus et al. 2019; Hamazaki et al. 2020; Zhong et al. 2024). The theoretical increase in power of the multi-population models compared to the single-population model can be explained by a drastic increase in sample size and a resulting lower SE of estimated SNP effects. However, in reality it is extremely unlikely that effects are exactly the same in all the combined populations, especially in the presented cases of population-specific SNPs. For such SNPs, we do not expect an increase in detection power using multi-population GWAS.

As expected, MP1 was highly influenced by the larger population when combining dataset of unequal sizes. Further, findings for both traits clearly demonstrated that cross-population genetic correlations differ from 1, making the assumptions of MP1 highly improbable. In contrast, the MP2 model tests for common effects between populations, and loosen the assumption of a trait being genetically identical in the combined populations. Because of more accurate model assumptions and equal representations of populations in significance tests, MP2 is expected to produce fewer type I errors than MP1 for the detection of shared MTAs.

### Multi-population GWAS identified multiple QTLs with common effects across populations

The MP2 combination of 6RW with other breeding populations detected five candidate QTLs for lodging and four additional non-overlapping candidate QTLs for heading date. Of these, one for heading date and two for lodging were not found when considering either of the populations alone. This can be explained by too little power to uncover small marker effects in the 6RW population, and alleles being too rare (MAC < 30) to pass the MAC filter in the other population(s). Previous studies in maize have also found that multi-population GWAS gave rise to many associations not found when studying the individual populations (Zuffo et al. 2022).

The additional candidate QTL detected in multi-population GWAS for heading date, reports a genomic region on chromosome 2H at 24.4–25.9 Mbp containing the *Ppd-H1* gene (Turner et al. 2005). When performing single-population GWAS, detection of the gene was limited to 2RW. This can be explained by the SNPs located within the *Ppd-H1* gene being close to monomorphic in the 2RS population (MAC of 1), very rare in the 6RS population (maximum MAC of 11) and no power to detect the region in the smaller populations i.e. 6RS and 6RW. *Ppd-H1* is the major gene determining long-day response in barley (Alqudah et al. 2020). It has functionally been validated in several studies, where mutations have been coupled to reduced response to long-day photoperiods (Turner et al. 2005; Parrado et al. 2023). Allelic variation has earlier been identified both within spring and winter populations, it is therefore expected that we can identify the gene when performing GWAS within 2RW (Alqudah et al. 2016; Digel et al. 2016). Besides detecting candidate QTLs that were not revealed by studying the involved populations separately, multi-population GWAS found that many of the candidate QTLs detected exclusively in single-population analyses for the 2RW, 6RS or 2RS populations, pointed to shared effects with the 6RW. Examples of such cases are the beforementioned region associated with *Ppd-H1*, *sdw1/denso*, as well as the lodging candidate QTL on chromosome 7H identified when performing GWAS within 6RS and by combining it with 6RW. The latter candidate QTL overlaps with a relatively large region found by Zhang et al. (2022) for stem related traits in barley. The identification of shared effects between populations is highly relevant, as QTL consistency provides evidence of shared additive genetics of the studied traits when comparing populations of different growth and row types. Further, it provides some validation of the identified QTLs, and ideally, if effects are large enough, it can provide inputs for marker-assisted selection (MAS) in an underpowered population, where no markers are detected by single-population GWAS. However, while this study detects several significant associations for both traits, the amount of variance explained by the individual SNPs were generally not large enough to be good candidates for MAS. Hence, a more suitable strategy for accelerating genetic improvement of lodging and heading date in the studied breeding population might be genomic selection.

### Some QTLs were population-specific

Consistent with earlier studies, not all candidate QTLs detected in single-population GWAS were detected in multi-population GWAS (Gebreyesus et al. 2019; Zuffo et al. 2022). Possible explanations are non-existing allelic variation of the QTLs in all combined populations, imperfect LD between QTLs and causal loci, or that the underlying genes have no effect on the phenotype in a given population due to interactions with the environments and/or epistasis (Legarra et al. 2021). Among the population-specific candidate QTLs that are undetected in multi-population GWAS is the heading date QTL found in population 2RS on chromosome 7H at 41.8 Mbp. The underlying gene (*Vrn-H3*) regulates the vernalization requirements in barley, and allelic variation typically differentiates spring and winter types of barley (Yan et al. 2006). Another 2RS-specific signal is the lodging-related candidate QTL on chromosome 4H at 19.1 Mbp, previously detected by Tsai et al. (2020) for its association with both lodging and straw breaking. This overlap is expected, as their study analysed a subset of the 2RS data we analysed (Tsai et al. 2020).

### Combining populations in GWAS comes with computational and statistical challenges

We combined up to four populations in joint GWAS. Theoretically, there are no limits to how many populations one can combine. However, as the number of combined datasets grows, the computational demands of MP2 becomes more resource-intensive, and the interpretation of the detected regions becomes harder. Here, we did not detect any new signals when performing multi-population GWAS across all four populations. This is expected, as few markers were common enough to pass the MAC filter of 10 in each population. Although QTLs were detected when applying the multivariate model to three and four populations, these were mostly a result of shared effects of two of the combined populations or a very large effect in the large 2RS population. However, we did observe a few cases of significant marker effects shared across all three populations including the association of heading date and the *Ppd-H1*-containing region, and the candidate QTL for lodging on chromosome 2H at position 650,512,028 bp explaining 13.8% of the genetic variation in 6RW.

### Conclusions and applications for future research

Overall, the study demonstrated that combining datasets from different breeding populations of barley can increase the power to detect GWAS associations. This proved particularly useful in identifying significant MTAs for the recently established 6RW population with limited data, where single-population GWAS struggled to detect significant associations.

For future applications of the MP2 model to more than two populations, we suggest focusing on differences in genetic effects between groups of populations. With the data presented here such an analysis could test for different marker associations between growth (spring versus winter) or row types (2-row versus 6-row).

Although we did not observe reverse phases between the studied populations, this could be different for other more diverse populations (Deng 2001; Lin et al. 2007; Teo et al. 2009). Our presented MP2 model can easily be modified to test for differences of scaled SNP regressions, and consequently allow identification of shared candidate QTLs with opposing signs in populations.

## Supplementary information

Supplementary figures S1-S9

Supplementary table legends

Table S1

Table S2

Table S3

Table S4

Table S5

Table S6

Table S7

Table S8

Table S9

Table S10

Table S11

Table S12

## Data Availability

All phenotype and genotype data used in this study have been deposited in DRYAD (10.5061/dryad.n2z34tn6h).
