# Correction: Multi-population GWAS detects robust marker associations in a newly established six-rowed winter barley breeding program

**DOI:** 10.1038/s41437-025-00751-3

**Published:** 2025-02-17

**Authors:** Cathrine Kiel Skovbjerg, Pernille Sarup, Ellen Wahlström, Jens Due Jensen, Jihad Orabi, Lotte Olesen, Just Jensen, Ahmed Jahoor, Guillaume Ramstein

**Affiliations:** 1 Nordic Seed A/S, Odder, Denmark; 2 Center for Quantitative Genetics and Genomics, Aarhus University, Aarhus C, Denmark; 3 Department of Plant Breeding, The Swedish University of Agricultural Sciences, Alnarp, Sweden

**Keywords:** Agricultural genetics, Population genetics, Genome-wide association studies, Rare variants, Plant breeding

Correction to: *Heredity* 10.1038/s41437-024-00733-x, published online 28 November 2024

In this article, Fig. 6 appeared in black/white, but should have appeared in colour.
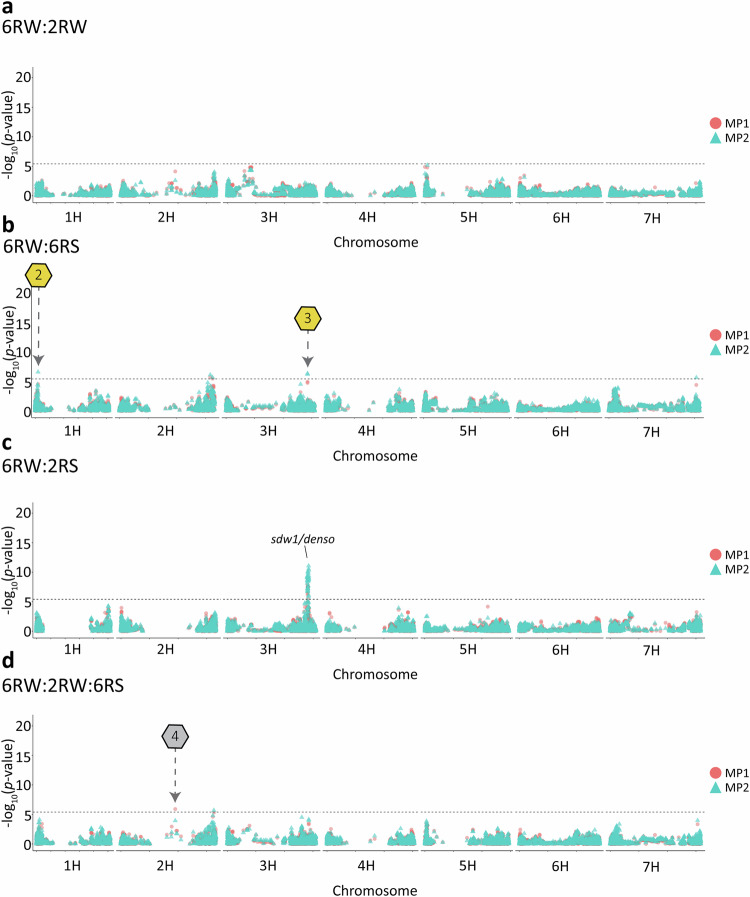


**Fig. 6 Overlaid Manhattan plot for lodging using multi-population GWAS models**. The red circles display the GWAS results from the univariate model (MP1) and the blue triangles display the results from the multivariate model (MP2). **a** 6RW:2RW. **b** 6RW:6RS. **c** 6RW:2RS. **d** 6RW:2RW:6RS. The horizontal dashed lines indicate the Bonferroni-corrected genome-wide significance threshold at −log_10_(*p*) = 5.4. Hexagons with numbers point towards candidate QTLs that were not found when performing single-population GWAS on the involved populations. Golden hexagons refer to high-confidence candidate QTLs found by the MP2 model alone or by both models. Candidate QTL 2 points to chromosome 1H at position 19,595,378 bp; Candidate QTL 3 points to chromosome 3H at position 570,634,774–570,635,897 bp; Candidate QTL 4 points to chromosome 2H at position 380,356,960 bp.

