# Supplementary material for: Multi-population GWAS detects robust marker associations in a newly established six-rowed winter barley breeding program
Source: Heredity (Edinb). 2024 Nov 28;134(1):33–48. doi: 10.1038/s41437-024-00733-x (PMC11724117; doi:10.1038/s41437-024-00733-x)
Supplement: Supplementary file 1 — Supplementary figures S1-S9 [file 41437_2024_733_MOESM1_ESM.docx]

**
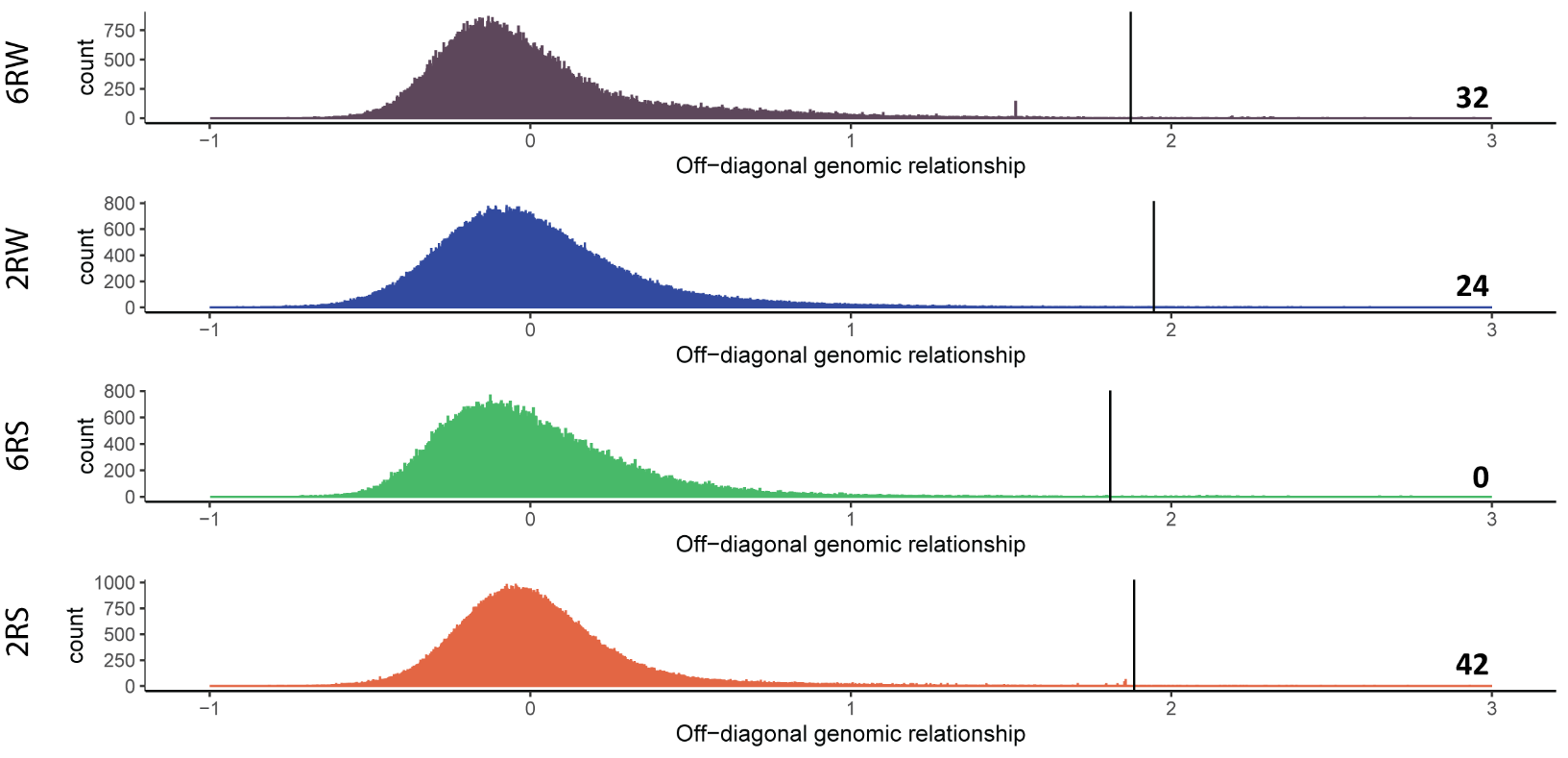
**

**Figure S1.** Histograms showing the genetic relationships among individuals within each of the four populations. Each histogram represents the distribution of genetic relatedness between pairs of individuals within a population, as measured by their off-diagonal elements of the genomic relationship matrix. The black vertical lines show the average diagonal element of the population-wise genomic relationship matrices for reference. Bold numbers in the lower right corner of each histogram denote the number of genotypes that have an off-diagonal relationship with other individuals exceeding 90% of their own self-relatedness (the diagonal).

**
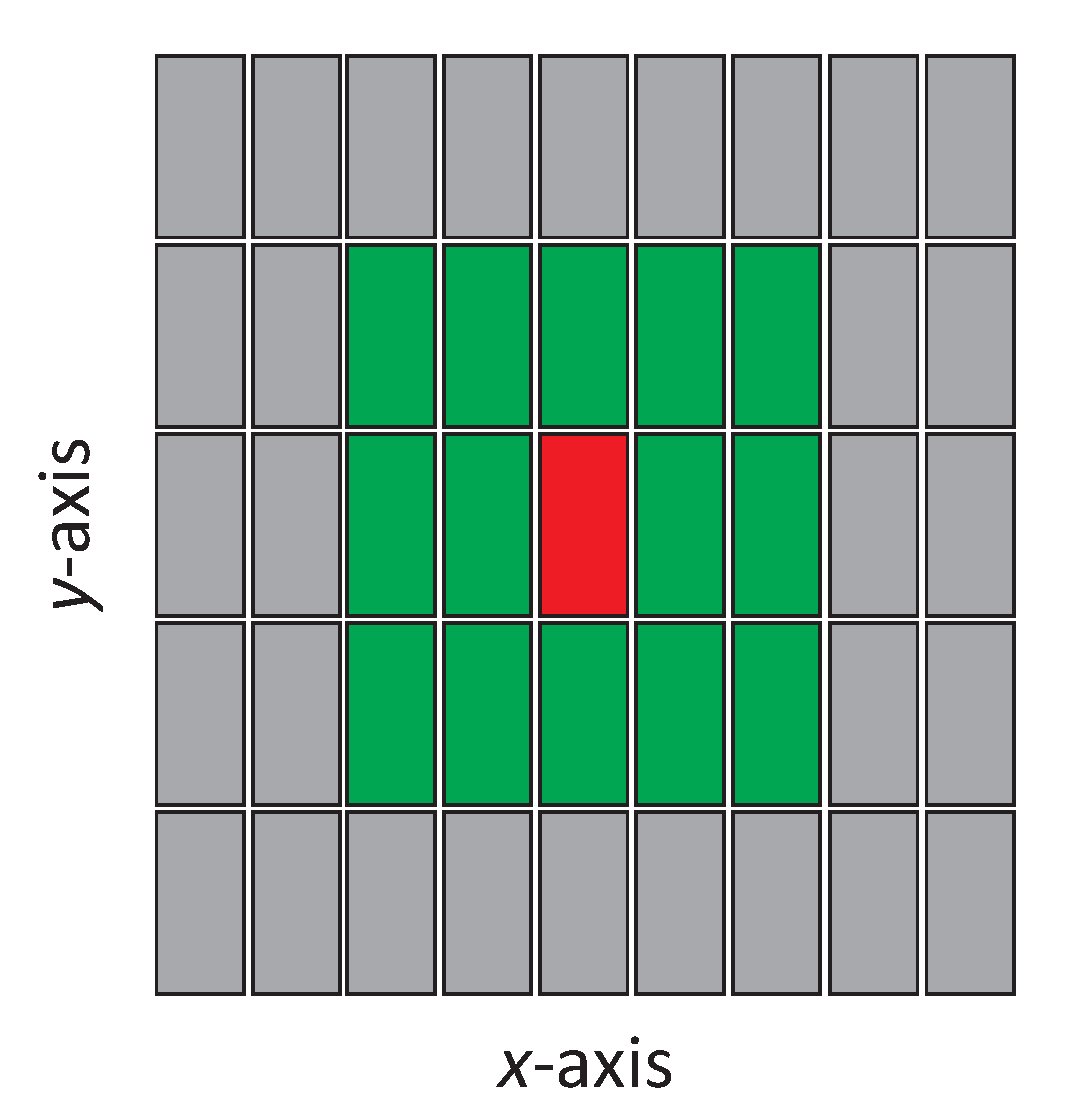
**

**Figure S2.** Explanation of the spatial effects integrated in the linear mixed models. For the data where plots contained both *x* and *y* field coordinates, the spatial effect of a target plot (red square) was fitted as a moving average including the 14 surrounding plots (green squares). As plots were generally larger in the direction of the *y*-axis than in the direction of the *x*-axis, we considered neighbouring plots to range from *x*-2 to *x*+2 and *y*-1 to *y*+1. The grey boxes indicate non-neighbouring plots that are not considered in the spatial effect of the target plot.


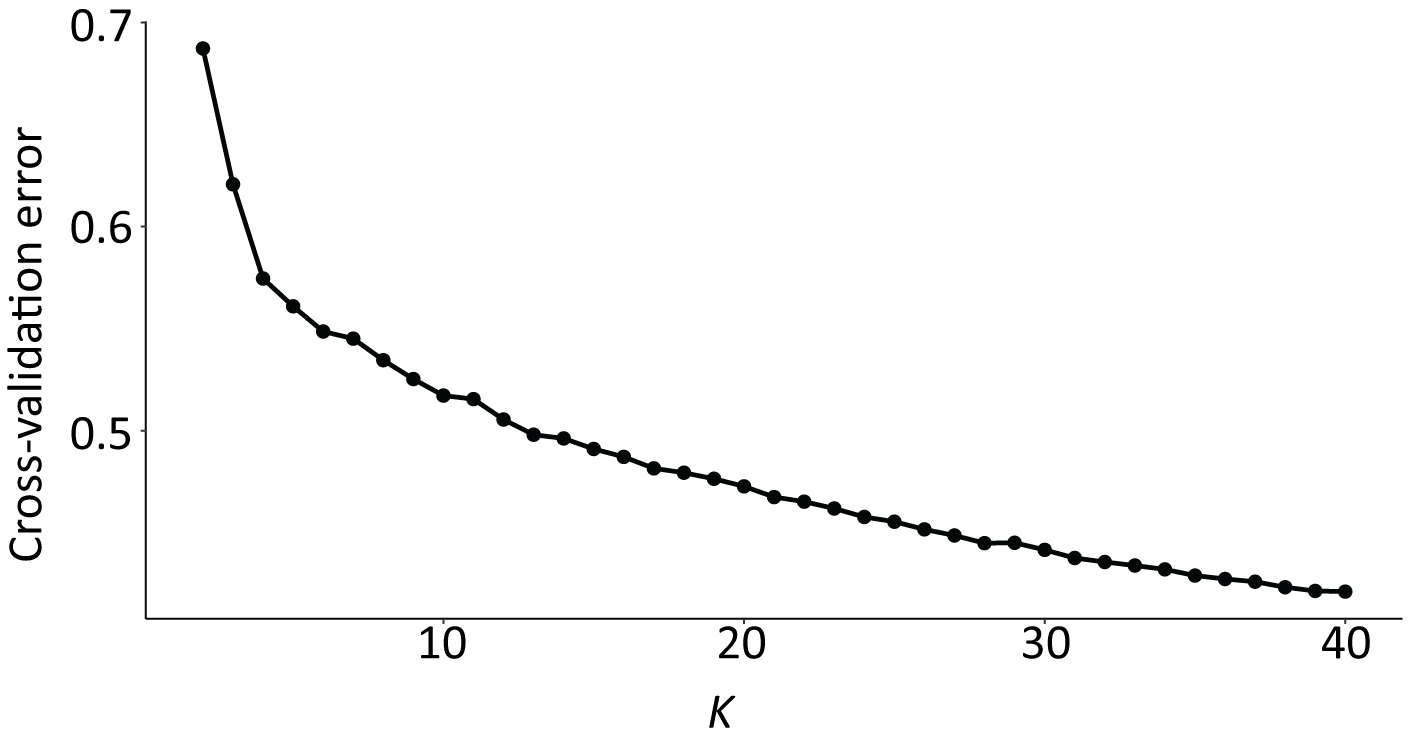


**Figure S3.** Cross-validation error of ADMIXTURE results with the number of ancestral populations (*K*) ranging from 2 to 40.


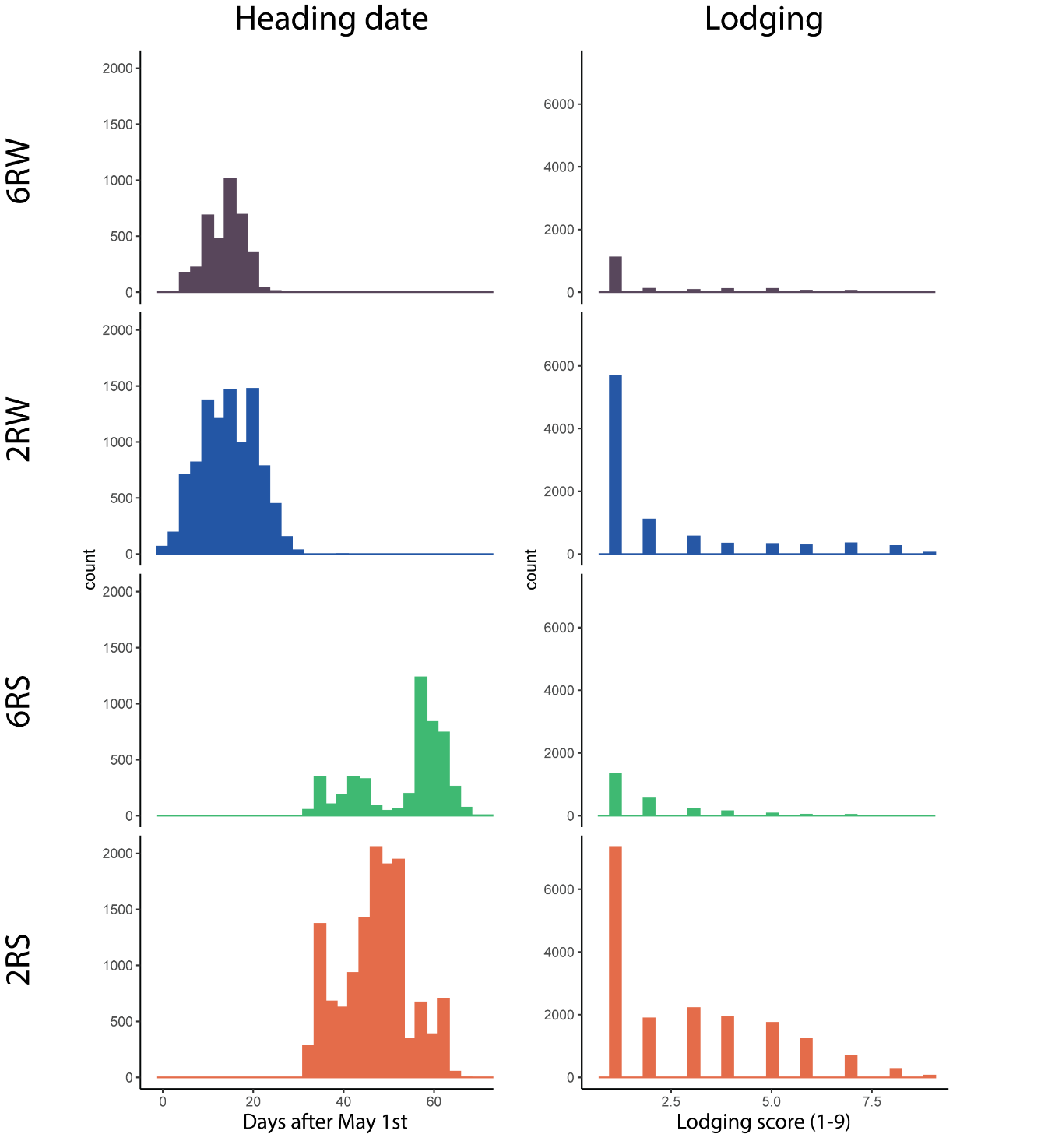


**Figure S4.** Phenotypic distribution of heading date and lodging across environments by populations. The 6RS population shows an additional peak for heading date due to significantly later sowing (approximately one month) in Finnish locations.

**
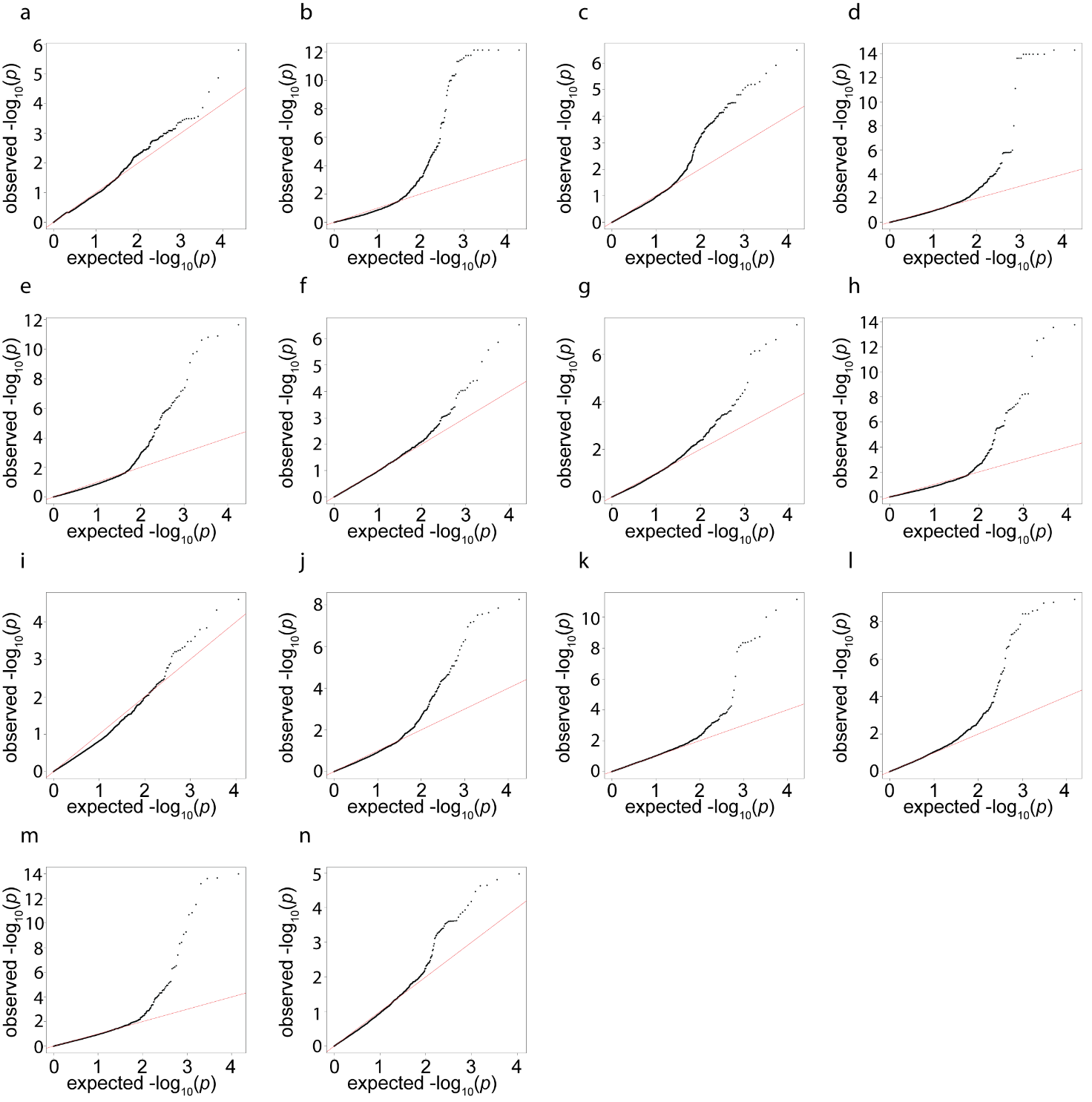
**

**Figure S5.** Quantile-quantile (QQ) plots for GWAS on heading date using the different single-population and multi-population models. Panels a-d show QQ-plots for single-population GWAS on the following populations: 6RW (a), 2RW (b), 6RS (c), and 2RS (d). Panels e-i display QQ-plots for the MP1 model applied to the following combinations of populations: 6RW:2RW (e), 6RW:6RS (f), 6RW:2RS (g), 6RW:2RW:6RS (h), and 6RW:2RW:6RS:2RS (i). Panels j-n display QQ-plots for the MP2 model applied to the following combinations of populations: 6RW:2RW (j), 6RW:6RS (k), 6RW:2RS (l), 6RW:2RW:6RS (m), and 6RW:2RW:6RS:2RS (n).

**
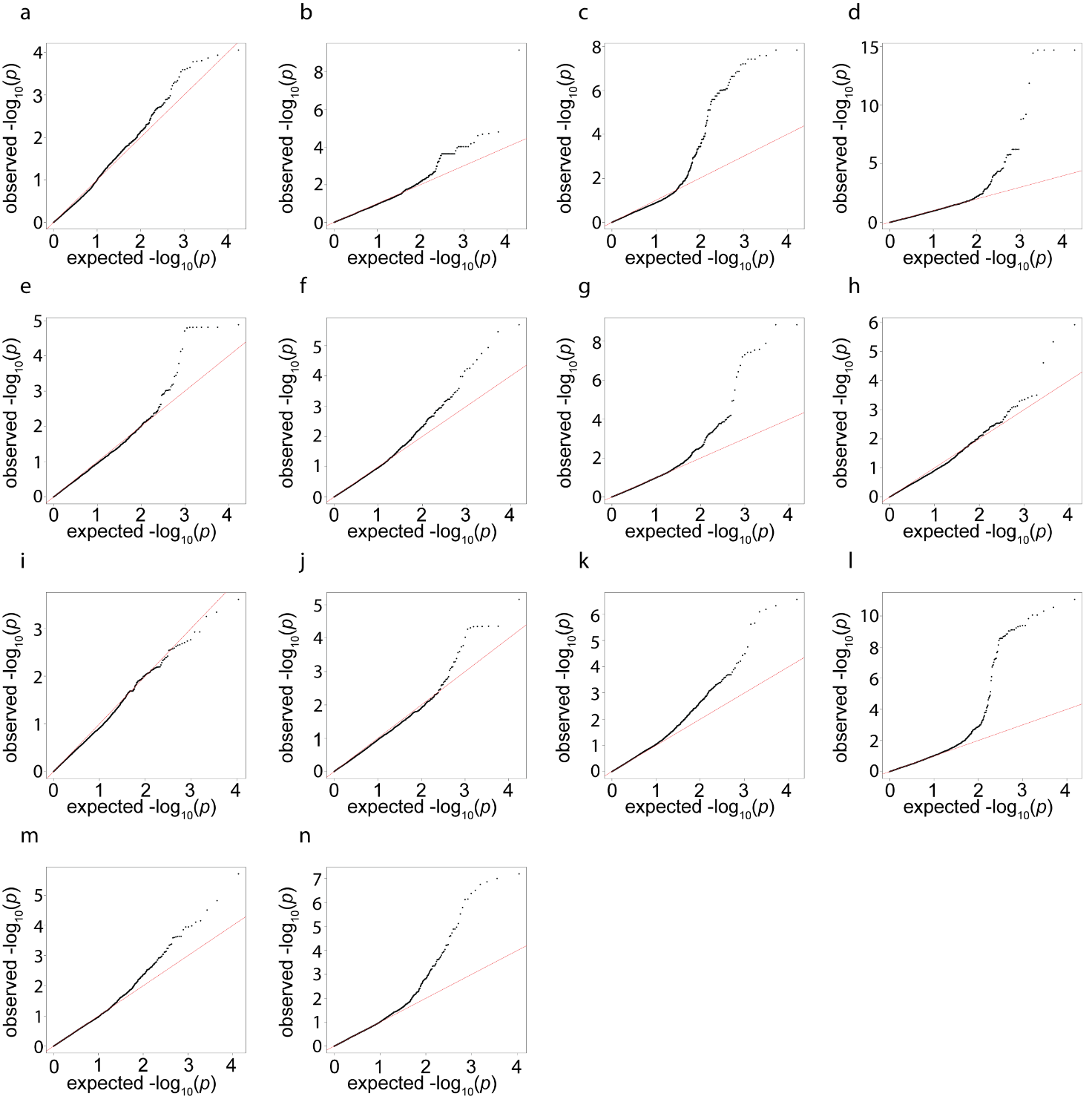
**

**Figure S6.** Quantile-quantile (QQ) plots for GWAS on lodging using the different single-population and multi-population models. Panels a-d show QQ-plots for single-population GWAS on the following populations: 6RW (a), 2RW (b), 6RS (c), and 2RS (d). Panels e-i display QQ-plots for the MP1 model applied to the following combinations of populations: 6RW:2RW (e), 6RW:6RS (f), 6RW:2RS (g), 6RW:2RW:6RS (h), and 6RW:2RW:6RS:2RS (i). Panels j-n display QQ-plots for the MP2 model applied to the following combinations of populations: 6RW:2RW (j), 6RW:6RS (k), 6RW:2RS (l), 6RW:2RW:6RS (m), and 6RW:2RW:6RS:2RS (n).


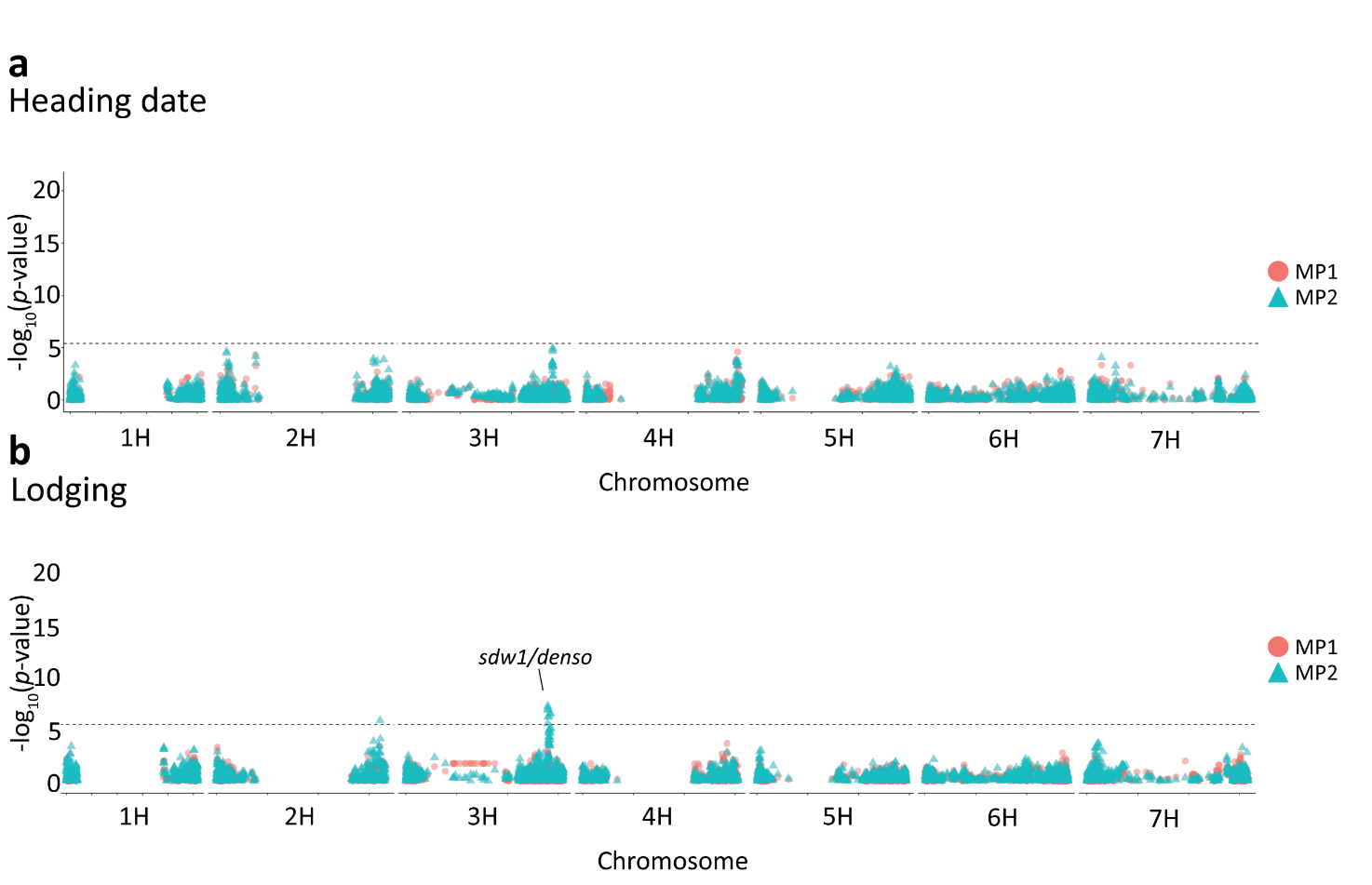
**Figure S7. Overlaid Manhattan plot for heading and lodging combining all four populations.** The red circles represent the GWAS results from the univariate model (MP1), while the blue triangles represent the results from the multivariate model (MP2). a) GWAS results for heading date b) GWAS results for lodging. The horizontal dashed lines indicate the Bonferroni-corrected genome-wide significance threshold at -log_10_(*p*)=5.4.


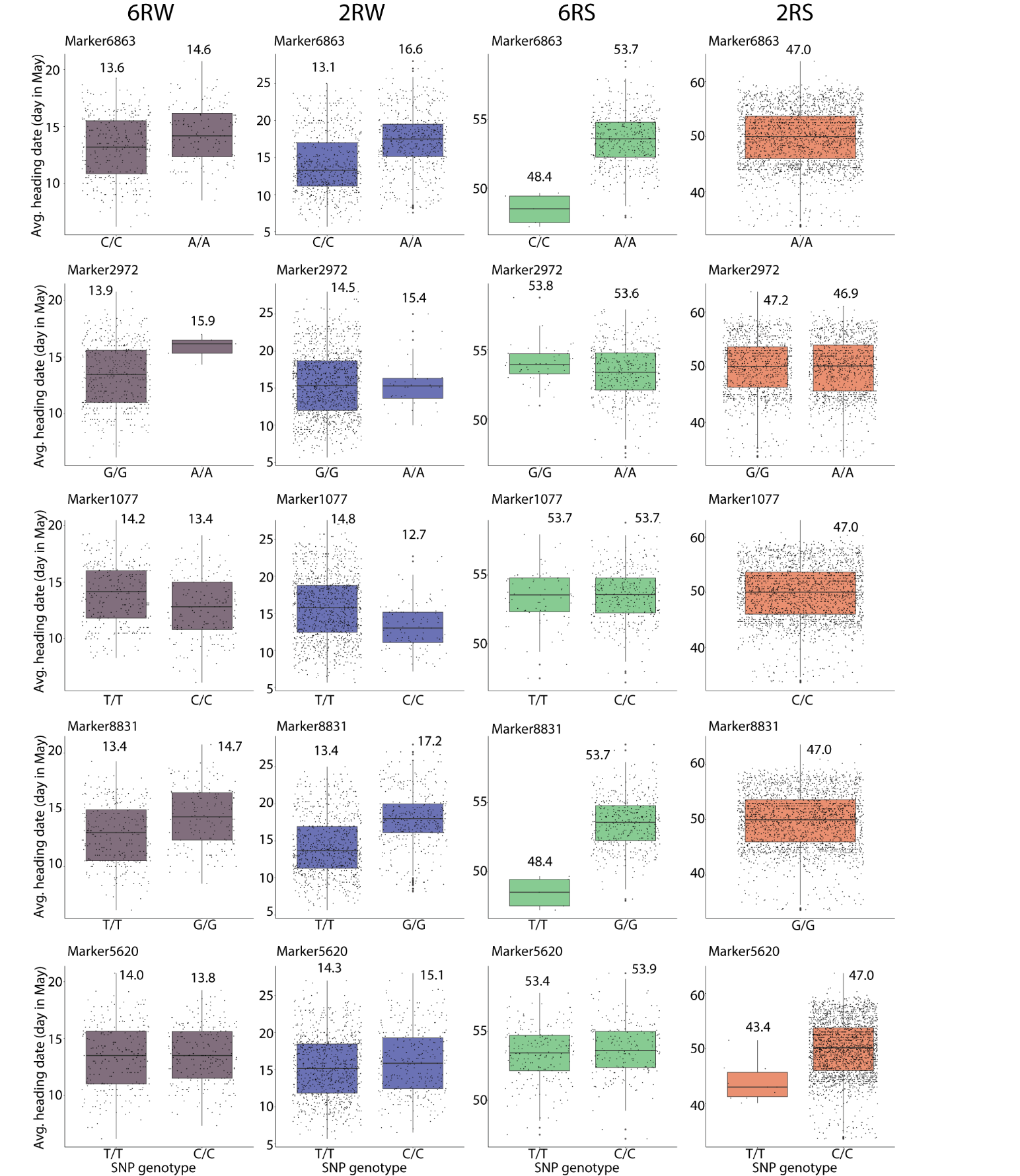


**Figure S8.** Boxplots for population-specific average heading date for lines across environments grouped by the genotype of the lead SNP of MP2-GWAS candidate QTLs. The numbers above the boxplots refer to the average phenotype of the given genotype. Breeding populations are stated in the top panel. The axis titles are displayed in the left margin (*y*) or at the bottom (*x*). To better visualize overlapping values on the *x*-axis, jitter has been applied.

**
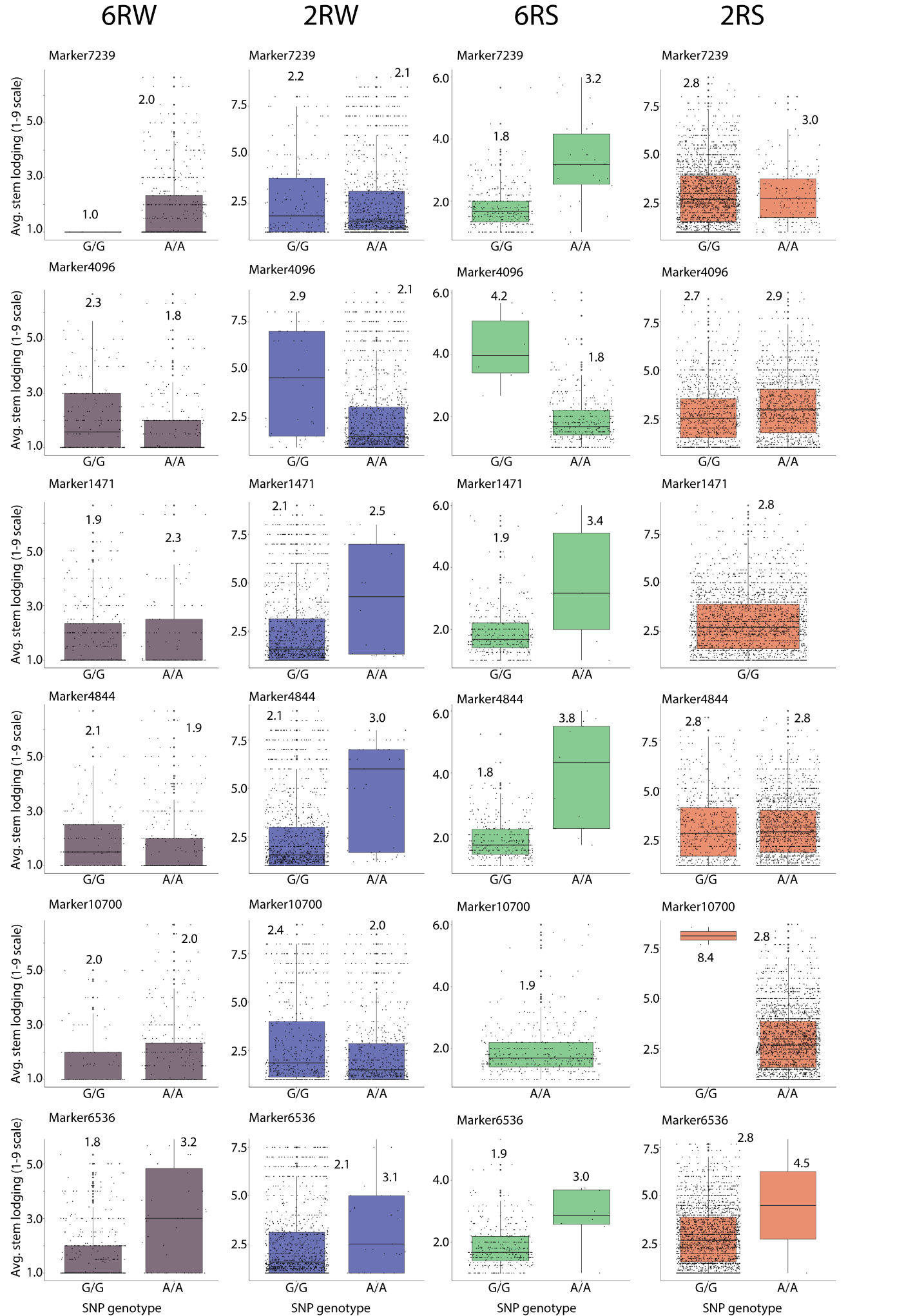
**

**Figure S9.** Boxplots for population-specific average lodging score for lines across environments grouped by the genotype of the lead SNP of MP2-GWAS candidate QTLs. The numbers above the boxplots refer to the average phenotype of the given genotype. Breeding populations are stated in the top panel. The axis titles are displayed in the left margin (*y*) or at the bottom (*x*). To better visualize overlapping values on the *x*-axis, jitter has been applied.
