# Supplementary material for: Multi-population GWAS detects robust marker associations in a newly established six-rowed winter barley breeding program
Source: Heredity (Edinb). 2024 Nov 28;134(1):33–48. doi: 10.1038/s41437-024-00733-x (PMC11724117; doi:10.1038/s41437-024-00733-x)
Supplement: Supplementary file 2 — Supplementary table legends [file 41437_2024_733_MOESM2_ESM.docx]

# **Supplementary Tables**

**Table S1.** Overview of field experiments per population.

**Table S2:** Scoring of stem lodging using a 1-9 scale.

**Table S3:** Descriptive statistics of SNP distribution and density.

**Table S4:** All significant MTAs identified in single-population GWAS.

**Table S5:** Trait correlations of heading date and lodging within populations.

**Table S6.** Genome-wide linkage phase similarities (LPS) between 6RW and 2RW in windows of 1 Mbp.

**Table S7.** Genome-wide linkage phase similarities (LPS) between 6RW and 6RS in windows of 1 Mbp.

**Table S8**. Genome-wide linkage phase similarities (LPS) between 6RW and 2RS in windows of 1 Mbp.

**Table S9.** Genome-wide linkage phase similarities (LPS) between 2RW and 6RS in windows of 1 Mbp.

**Table S10.** Genome-wide linkage phase similarities (LPS) between 2RW and 2RS in windows of 1 Mbp.

**Table S11.** Genome-wide linkage phase similarities (LPS) between 6RS and 2RS in windows of 1 Mbp.

**Table S12:** All significant MTAs identified in multi-population GWAS.
